# Inactivation of Intergenic Enhancers by EBNA3A Initiates and Maintains Polycomb Signatures across a Chromatin Domain Encoding CXCL10 and CXCL9

**DOI:** 10.1371/journal.ppat.1003638

**Published:** 2013-09-19

**Authors:** Marie L. Harth-Hertle, Barbara A. Scholz, Florian Erhard, Laura V. Glaser, Lars Dölken, Ralf Zimmer, Bettina Kempkes

**Affiliations:** 1 Department of Gene Vectors, Helmholtz Center Munich, German Research Center for Environmental Health, Munich, Germany; 2 Institut für Informatik, Ludwig-Maximilians-Universität München, München, Germany; 3 Department of Medicine, University of Cambridge, Cambridge, United Kingdom; Wistar Institute, United States of America

## Abstract

Epstein-Barr virus (EBV) causes a persistent infection in human B cells by establishing specific transcription programs to control B cell activation and differentiation. Transcriptional reprogramming of EBV infected B cells is predominantly driven by the action of EBV nuclear antigens, among them the transcriptional repressor EBNA3A. By comparing gene expression profiles of wt and EBNA3A negative EBV infected B cells, we have previously identified a broad array of cellular genes controlled by EBNA3A. We now find that genes repressed by EBNA3A in these cells are significantly enriched for the repressive histone mark H3K27me3, which is installed by Polycomb group (PcG) proteins. This PcG-controlled subset of genes also carries H3K27me3 marks in a variety of other tissues, suggesting that the commitment to PcG silencing is an intrinsic feature of these gene loci that can be used by EBNA3A. In addition, EBNA3A targets frequently reside in co-regulated gene clusters. To study the mechanism of gene repression by EBNA3A and to evaluate the relative contribution of PcG proteins during this process, we have selected the genomic neighbors *CXCL10* and *CXCL9* as a model for co-repressed and PcG-controlled genes. We show that EBNA3A binds to CBF1 occupied intergenic enhancers located between *CXCL10* and *CXCL9* and displaces the transactivator EBNA2. This impairs enhancer activity, resulting in a rapid transcriptional shut-down of both genes in a CBF1-dependent manner and initiation of a delayed gain of H3K27me3 marks covering an extended chromatin domain. H3K27me3 marks increase gradually and are maintained by EBNA3A. Our study provides direct evidence that repression by EBNA3A requires CBF1 and that EBNA3A and EBNA2 compete for access to CBF1 at identical genomic sites. Most importantly, our results demonstrate that transcriptional silencing by EBNA3A precedes the appearance of repressive PcG marks and indicate that both events are triggered by loss of enhancer activity.

## Author Summary

Epstein-Barr virus (EBV) is a γ-herpesvirus which establishes a latent infection in human B cells and is associated with the pathogenesis of several types of cancer. Here, we report that cellular genes repressed by the EBV nuclear antigen 3A (EBNA3A) in EBV infected B cells frequently form contiguous clusters in the human genome and are committed to epigenetic silencing by Polycomb group (PcG) proteins. The chemokine genes *CXCL10* and *CXCL9* and their receptors on NK and T cells are critical weapons of the infected host to control herpesvirus infections. *CXCL10* and *CXCL9* are close neighbors within an extended PcG-controlled domain. We show that EBNA3A binds to intergenic enhancers located between *CXCL10* and *CXCL9* and displaces the transactivator EBNA2. This process impairs enhancer activity, resulting in a rapid transcriptional shut-down of both genes followed by a delayed gain of PcG histone marks. These PcG marks increase within the following weeks and are maintained by EBNA3A. Our results show that rapid transcriptional shut-down of distal genes and domain-wide PcG silencing is triggered by loss of enhancer activity and suggest that EBNA3A can reprogram the cellular genome in order to escape the immune surveillance of the host.

## Introduction

Epstein Barr virus (EBV) is a ubiquitous human herpesvirus that establishes a persistent latent infection in more than 90% of the adult human population. EBV can cause infectious mononucleosis and is associated with the pathogenesis of endemic Burkitt's lymphoma, Hodgkin lymphoma, lymphoproliferative disorders in immuno-suppressed and HIV-infected people, as well as epithelial malignancies [1]–[3]. EBV infection of primary human B cells causes cell cycle entry of the infected cells. This process is controlled by the concerted action of 6 latent EBV nuclear antigens (EBNAs) and 3 latent membrane proteins (LMPs), which mimic cellular functions required for B cell proliferation and differentiation [3], [4]. *In vivo*, the latent viral gene expression program is dynamic. It switches to at least two additional distinct viral gene expression patterns (latency I and II), which reprogram the differentiation state of the infected host B cells to finally become resting memory B cells that serve as a life-long reservoir for the virus [5]. *In vitro*, the infected B cells convert into permanently proliferating lymphoblastoid B cell lines (LCLs), which phenocopy activated B cell blasts and are frozen at that state of differentiation as long as all 9 latent proteins are expressed (latency III).

*EBNA3A* is a member of the *EBNA3* gene family consisting of *EBNA3A*, *-3B*, and *-3C*, which is expressed during latency III. The EBNA3A and -3C proteins are transcriptional regulators that exhibit robust repressor activity when tethered to DNA [6]–[8] and can interact with cellular factors involved in transcriptional regulation, including the co-repressor CtBP and histone deacetylases [9]–[12]. Importantly, all of the EBNA3 proteins as well as the transactivator EBNA2 are invariably co-expressed in established LCLs and bind to the cellular CBF1 protein (C promoter binding factor, also known as CSL, Suppressor of Hairless, Lag-1 or RBPJ). CBF1 is a ubiquitous sequence specific DNA-binding factor which recruits co-repressor complexes to *cis*-regulatory elements. CBF1 is highly conserved throughout evolution and constitutes the major DNA-adaptor of activated NOTCH that regulates many aspects of metazoan development and tissue renewal [13]. Like the cellular NOTCH protein, the viral EBNA2 protein binds to CBF1, displaces the co-repressor complex and activates transcription. Since EBNA3 proteins can interfere with CBF1-dependent EBNA2 transactivation of viral promoters in reporter gene assays, it has been suggested that EBNA3 proteins antagonize EBNA2 functions [14]–[18]. A recent ChIP-seq study for EBNA2 and CBF1 in LCLs reported predominant binding of both proteins in the cellular genome at distal intergenic and intronic enhancers [19], which were defined by characteristic chromatin signatures [20]–[22]. These enhancers bound by EBNA2 have long-range interactions with promoters of EBNA2 up-regulated genes, such as *MYC* [19]. In fact, EBNA3A antagonizes EBNA2 activation of *MYC* [23]. However, reciprocal binding of EBNA2 or EBNA3A to CBF1 occupied genomic sites has not been shown until now.

By comparing gene expression profiles of EBNA3A proficient and deficient LCLs, we recently identified a broad array of EBNA3A controlled cellular genes which have a significant impact on the cellular phenotype. Comparison of EBNA3A and EBNA2 target genes indicated that the two proteins might indeed counter-regulate a significant set of cellular genes [24]. To date, a number of microarray studies have been undertaken to investigate the impact of EBNA3 proteins on host gene expression [25]–[30]. These studies uncovered an extensive cooperation between the EBNA3 proteins in the regulation of host genes. Importantly, gene repression by EBNA3 proteins, in particular EBNA3C, correlates with increased trimethylation of histone H3 at lysine 27 (H3K27me3), suggesting a role for Polycomb group (PcG) proteins in the repression of host genes [27], [29]–[33].

PcG proteins are epigenetic regulators that maintain the tissue specific gene expression program, which is set during development and differentiation, and hence retain cellular memory. In mammals, two major PcG complexes, Polycomb repressive complex 1 and 2 (PRC1 and PRC2), have been characterized. Trimethylation of H3K27 constitutes a key feature of PcG-silenced chromatin and is catalyzed by PRC2 that contains the histone methyltransferase EZH2 and the essential cofactor SUZ12. PRC1 can bind to H3K27me3 and catalyzes histone H2A ubiquitylation [34], [35]. PcG recruitment to mammalian genomic elements is not well understood. So far, two DNA sequence elements [36], [37], CpG islands and GC-rich sequences [38]–[40], as well as non-coding RNAs were reported to recruit PcG complexes [35]. More recently, enhancers have been functionally linked to the PcG silencing system and were shown to regulate PcG patterns on distal genes [41], [42].

The goal of this study was to analyze the mechanism of gene repression by EBNA3A and to evaluate the relative contribution and dynamics of PcG protein engagement during this process. To this end, the EBNA3A repressed genes described previously [24] were analyzed *in silico* for H3K27me3 occupancy and their genomic positions using ENCODE data sets [43], [44]. We found that EBNA3A repressed genes are predominantly silenced by PcG proteins and are significantly enriched in co-regulated gene clusters. The EBNA3A target genes *CXCL10* and *CXCL9* were chosen as a model since they exhibit both features: They are co-regulated genomic neighbors within an extended PcG-controlled domain. Both encode interferon (IFN) inducible T cell attracting chemokines [45]. Their repression by EBNA3A might thus counteract antiviral responses of the cell and promote essential steps of the viral life cycle in the infected host. Interestingly, we found that EBNA3A antagonizes IFN mediated induction of *CXCL10/9*, indicating that it acts on a dominant master control region of the *CXCL10/9* domain. By using EBNA3A conditional and CBF1 negative B cell lines, we showed that repression by EBNA3A requires CBF1 and explored the kinetics of transcriptional repression and the deposition of chromatin marks. We found that H3K27me3 marks spread across the *CXCL10/9* domain subsequent to transcriptional down-regulation of the genes. Our data indicate that both events are a consequence of the binding of EBNA3A to CBF1 occupied intergenic enhancers that are otherwise bound and maintained in an active state by EBNA2 in lymphoblastoid B cells.

## Results

### EBNA3A repressed genes are frequently silenced by PcG proteins and form contiguous clusters in the human genome

**Figure 1:**
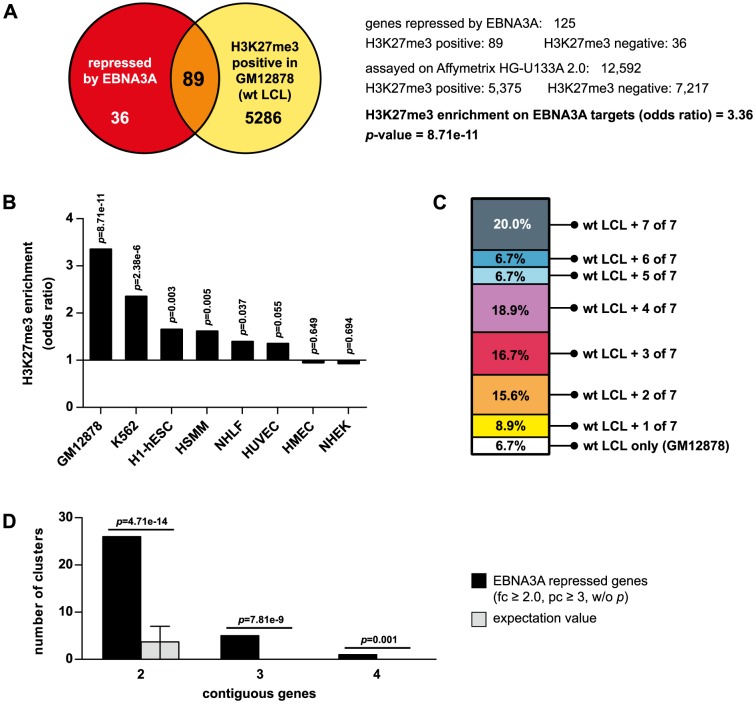
EBNA3A repressed genes are significantly enriched for genes controlled by PcG proteins in diverse tissues and frequently form contiguous gene clusters in the human genome. (A) Overlap of ENCODE H3K27me3 ChIP-Seq data in wt LCLs (GM12878) with NM genes represented on Affymetrix HG-U133A 2.0 microarrays used in our previous expression profiling study. Enrichment for H3K27me3 was calculated for 125 cellular genes repressed by EBNA3A ≥2.0-fold (*p*≤0.05) when compared to the total number of genes analyzed on the array. Enrichment is given as odds ratio and was tested for statistical significance using Fisher's exact test. (B) H3K27me3 marks at EBNA3A repressed genes are not only present in LCLs. The 125 EBNA3A repressed genes were analyzed for H3K27me3 occupancy in 7 EBV negative cell lines of various tissue origin using ENCODE data. Odds ratios were calculated with respect to the H3K27me3 status of the total number of genes analyzed on HG-U133A 2.0 arrays in the individual cell lines and tested for statistical significance with Fisher's exact test. (C) EBNA3A repressed genes are committed to PcG control in various cell lines. The 89 EBNA3A repressed genes that scored positive for H3K27me3 in wt LCLs were analyzed for the proportion of genes that are also silenced by H3K27me3 in EBV negative cell lines. Results indicate the percentage of EBNA3A targets detected as PcG-silenced in LCLs only or in addition in some of the 7 analyzed cell lines. (D) EBNA3A repressed genes frequently form co-regulated gene clusters in the human genome. 220 EBNA3A repressed genes were filtered from previous expression profiling data by applying the indicated thresholds for fold change (fc), present call (pc), and *p*-value criteria and analyzed for their position within the human genome. 47 genes could be assigned to groups of 2, 3, and 4 gene clusters. The expected frequency of clustered genes in the same number of randomly selected genes was calculated for comparison. Error bars indicate 90% confidence intervals (binomial test). Please note that genes in groups overlap, e.g., a cluster of 3 contiguous genes includes the clusters of 2 contiguous genes.

Previously it has been reported that the EBNA3A repressed cellular genes *CDKN2A* (referred as *p16* in the following), *BCL2L11* (referred as *BIM* in the following), *TOX*, and *NOTCH2* exhibit elevated levels of PRC2 signatures [27], [31]–[33]. To evaluate if PcG silencing is a common feature of EBNA3A repression, we analyzed our set of EBNA3A repressed genes [24] for H3K27me3 occupancy in EBV infected B cells. The analysis was performed on 125 genes repressed by EBNA3A at least 2-fold (*p*≤0.05), which have been identified by comparing gene expression profiles of EBNA3A proficient and deficient LCLs (referred as wt and EBNA3A negative LCLs in the following). Genomic sections from position −500 bp relative to the transcription start site (TSS) till the end of the respective genes were screened for H3K27me3 positive segments using H3K27me3 ChIP-seq data provided by the ENCODE Consortium for the wt LCL GM12878 [44]. In our Affymetrix gene array analysis a total of 12,592 genes had been analyzed. 5,375 (43%) of these carried the H3K27me3 signature in GM12878 cells. We found that the majority of EBNA3A repressed genes, 89 of 125 (71%), scored positive for this PRC2-catalyzed histone mark. Thus, compared to the total number of genes represented on the array, PcG signatures were significantly enriched within the set of EBNA3A repressed genes (odds ratio of 3.36; *p* = 8.71e-11) (Figure 1A). Hence, trimethylation of H3K27 is a very common feature of EBNA3A silenced genes.

The *p16* locus was shown to be enriched for H3K27me3 in wt LCLs compared to EBNA3A negative LCLs [33] but is a well-known PcG-controlled locus irrespective of EBV infection and tissue origin [46], [47]. Thus, we speculated that H3K27me3 positive EBNA3A repressed genes are generally committed to PcG control even in EBV negative cells. To test this hypothesis, the H3K27me3 status of the 125 genes repressed by EBNA3A in LCLs was surveyed in 7 EBV negative cell lines of distinct tissue origin using ENCODE data. Indeed, significant enrichment of H3K27me3 on the 125 genes was also detected in 5 of the 7 EBV negative cell lines and absent only in the 2 cell lines HMEC and NHEK (Figure 1B). Across all cell lines tested, only a minority of H3K27me3 marks were exclusively seen on EBNA3A targets in LCLs (6.7%). In fact, 93% of the EBNA3A repressed genes that scored positive for H3K27me3 in LCLs were also silenced by PcG mechanisms in at least one additional cell line (Figure 1C). These findings suggest that EBNA3A is one of multiple direct or indirect triggers that induce PcG silencing and that PcG silencing might be an inherent capacity of the gene locus.

Upon closer inspection of EBNA3A repressed genes, we realized that co-regulated genes frequently formed contiguous clusters which were not interrupted by other annotated genes. A bioinformatics analysis of the genomic positions of all genes repressed by EBNA3A at least 2-fold revealed that 47 of 220 genes were grouped in clusters of 2, 3 and even 4 co-regulated genes (Figure 1D). Cluster arrangement of the EBNA3A repressed genes was highly significant when compared to randomly selected genes (Figure 1D). Thus, EBNA3A might program the host expression profile by controlling co-regulated gene clusters in addition to controlling individual target genes.

### *CXCL10* and *CXCL9* are neighbors in an extended PcG-controlled domain, strongly expressed in EBNA3A negative LCLs and rapidly repressed upon EBNA3A expression

**Figure 2:**
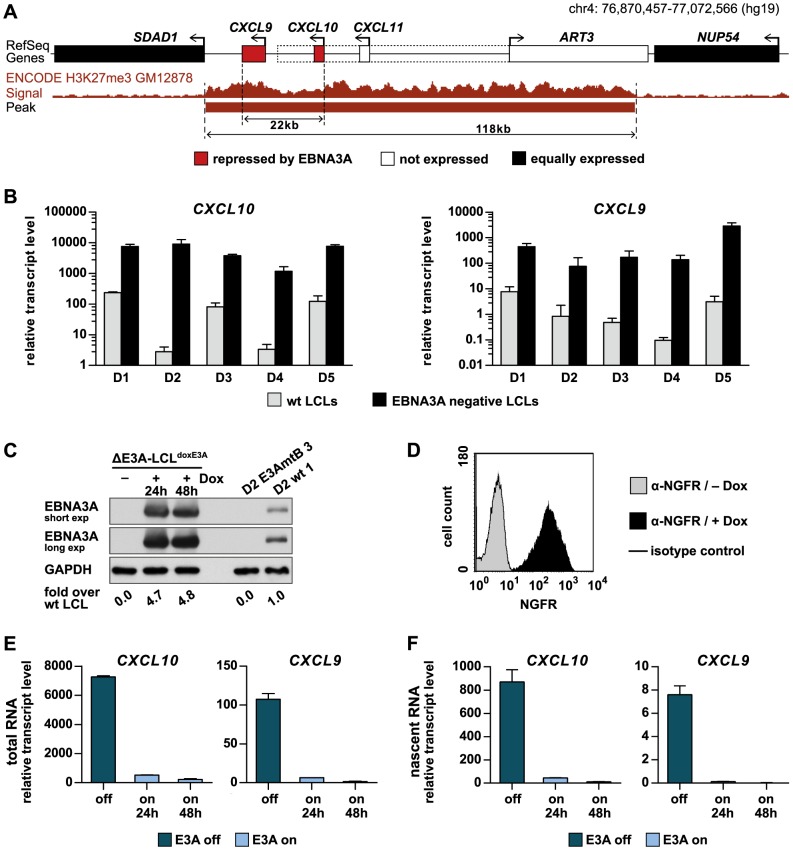
*CXCL10* and *CXCL9* reside within a PcG-controlled chromatin domain of 118 kb and are rapidly repressed upon EBNA3A expression. (A) Schematic representation of a genomic region on human chromosome 4 showing the location of the EBNA3A repressed genes *CXCL10* and *CXCL9* as well as flanking genes and the H3K27me3 coverage in wt LCLs (GM12878) according to ENCODE data. *CXCL10* and *9* comprise a region of 22 kb, which is embedded in an H3K27me3 positive domain of 118 kb. *CXCL11* and *ART3* also reside within this domain but are neither expressed in wt nor EBNA3A negative LCLs, while *SDAD1* and *NUP54* show similar expression levels irrespective of the EBNA3A status. Dotted lines demarcate an alternative TSS of *ART3*, which is not used in LCLs. (B) Validation of differential *CXCL10* and *9* expression in wt and EBNA3A negative LCLs derived from 5 unrelated B cell donors. Transcripts of *CXCL10* and *9* were quantified by qPCR in triplicate cDNA preparations from LCLs established by infection of B cells with EBVwt or either EBV-E3AmtA (D1, D4, D5) or EBV-E3AmtB (D2, D3). Data were normalized to 18S rRNA levels and are given as mean ± standard deviation (SD). (C) Western blot analysis of EBNA3A expression in ΔE3A-LCL^doxE3A^ cells prior to and 24 or 48 h post treatment with 100 ng/ml Dox. Protein extracts of the parental EBNA3A negative LCL (D2 E3AmtB 3) and a corresponding wt LCL (D2 wt 1) served as a negative and positive control, respectively. GAPDH immunodetection was used as loading control. Protein band intensities were quantified by densitometry. EBNA3A protein levels were normalized to GAPDH and are given as x-fold expression relative to the expression level in the corresponding wt LCL. (D) Flow cytometric analysis of NGFR expression in ΔE3A-LCL^doxE3A^ cells prior to and 24 h post treatment with 100 ng/ml Dox. Staining of cells with isotype-matched nonspecific antibodies served as a negative control. (E) EBNA3A induction in conditional LCLs rapidly down-regulates *CXCL10* and *9* expression. ΔE3A-LCL^doxE3A^ cells were induced for EBNA3A (E3A) expression by treatment with 100 ng/ml Dox for 24 or 48 h or left untreated. For metabolic labeling of nascent RNA, cells were cultured in the presence of 4sU for 1 h prior to harvesting. *CXCL10* and *9* transcripts in total RNA were quantified by qPCR, normalized to total 18S rRNA levels, and are given as mean ± SD of two biological replicates analyzed in triplicates. (F) *CXCL10* and *9* repression by EBNA3A is achieved by reduction of *de novo* transcription. Nascent RNA was isolated from total RNA prepared in (E). Nascent *CXCL10* and *9* transcripts were quantified by qPCR, normalized to nascent 18S rRNA levels, and are given as mean ± SD of two biological replicates analyzed in triplicates.

To investigate the mechanism of gene repression by EBNA3A, we decided to focus on a representative EBNA3A repressed locus which exhibits both features, repression by PcG proteins and co-repression of a gene cluster. We chose the genes *CXCL10* and *CXCL9*, which encode for T cell chemoattractants, are close neighbors on chromosome 4, and are co-repressed by EBNA3A. These two genes reside within a chromatin domain of 118 kb that scores positive for H3K27me3 in wt LCLs (Figure 2A). *CXCL11* and *ART3* also map to this domain but are neither expressed in wt nor EBNA3A negative LCLs. According to our expression profiling data, the genes flanking the 118 kb H3K27me3 domain, *SDAD1* and *NUP54*, are equally well expressed in wt and EBNA3A negative LCLs [24]. By quantitative (q) PCR analysis we confirmed elevated transcript levels of *CXCL10* and *9* in EBNA3A negative compared to wt LCLs derived from 5 unrelated B cell donors (Figure 2B). *CXCL10* expression substantially exceeded expression levels of *CXCL9* in all samples (Table S1). Low *CXCL11* transcript levels were detected in EBNA3A negative LCLs and further reduced in wt LCLs, while *ART3* transcripts could not be detected (Table S1).

To analyze the repression kinetics of *CXCL10* and *9*, we generated LCLs that express EBNA3A in a doxycycline (Dox) dependent manner (ΔE3A-LCL^doxE3A^). To this end, a previously described EBNA3A negative LCL [24] was stably transfected with an episomal vector system [48] that drives the simultaneous expression of EBNA3A and a truncated version of the NGF receptor (NGFR) upon Dox treatment. In the absence of Dox, EBNA3A was not expressed in ΔE3A-LCL^doxE3A^ cells (Figure S1A), while increasing amounts of Dox induced EBNA3A transcript and protein in a dose-response relationship (Figure S1B, C). In all subsequent experiments cells were treated with 100 ng/ml Dox. According to our western blot analysis, this treatment induced EBNA3A protein levels that were 5 times higher than EBNA3A levels seen in the corresponding wt LCL (Figure 2C). NGFR was expressed by the entire Dox-treated cell population with some variance in expression levels as determined by flow cytometric analysis at the single-cell level (Figure 2D). Upon EBNA3A induction, transcript levels of *CXCL10* and *9* rapidly dropped within 24 hours (h) and further declined during the next 24 h (Figure 2E).

In theory, EBNA3A could down-regulate steady state mRNA levels of *CXCL10* and *9* by either reducing transcription rates or mRNA stability. To test whether EBNA3A reduces transcription of the two genes, we metabolically labeled nascent RNA with the nucleoside analogue 4-thiouridine (4sU). 4sU is incorporated into mRNA during transcription [49] and can be biotinylated *in vitro*. The biotin tag is then used to separate nascent RNA from untagged pre-existing RNA by streptavidin beads [50]. Nascent *CXCL10* and *9* transcripts were quantified prior to and 24 or 48 h post EBNA3A induction. The EBNA3A-dependent decrease of *CXCL10* and *9* transcripts in nascent RNA preparations mirrored the results observed with total RNA preparations (Figure 2F). Hence, repression of both genes by EBNA3A is achieved by reduction of *de novo* transcription.

### Down-regulation of *CXCL10* and *CXCL9* transcription precedes the gain of repressive H3K27me3 chromatin marks

**Figure 3:**
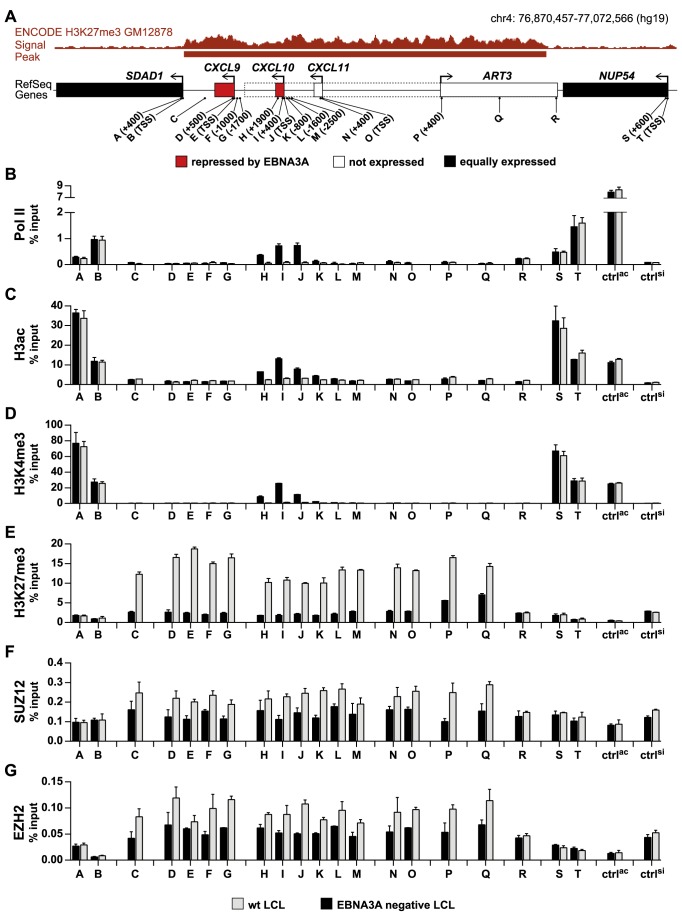
H3K27me3 marks are elevated throughout the *CXCL10/9* domain in EBNA3A positive LCLs. (A) Schematic representation of the *CXCL10* and *9* encompassing domain indicating the positions of primer pairs A-T used for qPCR quantification of ChIPed DNA relative to the TSS of the analyzed genes. (B–G) ChIP analysis of established wt and EBNA3A negative LCLs (D2 wt 1 and D2 E3AmtB 3) showing the abundance of (B) Pol II, (C) H3ac, (D) H3K4me3, (E) H3K27me3, (F) SUZ12, and (G) EZH2. Bars indicate the enrichment of Pol II, of histone modifications and of PRC2 subunits at the individual loci as assessed by qPCR with primer pairs A-T. Primer pairs for the TSS of *GAPDH* (ctrl^ac^) and a pericentromeric region on chromosome 1 (ctrl^si^) were included as a control for active and silenced chromatin, respectively. Bar height was calculated as percentage of ChIPed DNA recovered from input DNA, after subtraction of values from negative control IgG precipitation. Data are representative of three independent experiments. Error bars indicate SD of triplicate qPCR reactions (with exception of data in panel G, which are given as mean ± range of two independent experiments).

To determine whether repression of *CXCL10* and *CXCL9* correlates with deposition of the repressive H3K27me3 mark, chromatin immunoprecipitation (ChIP) analyses for established wt and EBNA3A negative LCLs were performed. In addition, RNA polymerase II (Pol II) occupancy and the abundance of two distinct modifications of histone H3 that are associated with the active state of genes, namely acetylation (H3ac) and trimethylation of lysine 4 (H3K4me3), were analyzed. ChIP analyses were not restricted to the *CXCL10* and *9* genes but extended across the entire putative domain as well as to flanking genes by using primer pairs designated A-T (Figure 3A). Pol II, H3ac, and H3K4me3 levels were elevated across the *CXCL10* gene in EBNA3A negative LCLs consistent with its high expression in these cells (Figure 3B, C, D). The lack of these marks at the *CXCL9* locus might be caused by the considerably lower expression levels of *CXCL9* compared to *CXCL10* (Table S1). Repression of *CXCL10* and *9* in wt LCLs correlated with clearly increased levels of H3K27me3 histone marks across the entire chromatin domain of 118 kb (primer pairs C-Q) (Figure 3E). In contrast, neither chromatin modifications nor Pol II occupancy were affected by EBNA3A when genomic positions outside of the domain were analyzed (primer pairs A, B, R, S, T, ctrl^ac^, ctrl^si^). These results were confirmed in ChIP analyses performed with another independent pair of wt and EBNA3A negative LCLs (Figure S2). Trimethylation of H3K27 is catalyzed by PRC2 that contains the histone-lysine methyltransferase EZH2 and the essential cofactor SUZ12 within its core [34]. Elevated levels of both proteins were detected along with H3K27me3 across the entire chromatin domain in wt compared to EBNA3A negative LCLs (Figure 3F, G). In summary, the data indicate that EBNA3A forces the accumulation of PRC2 and the resulting catalysis of H3K27me3 across the *CXCL10/9* domain. According to our expression profiling data, neither PRC2 subunits nor the H3K27 demethylases UTX and JMJD3 were differentially expressed between wt and EBNA3A negative LCLs (Table S2 and [24]). In addition, protein levels of PRC2 subunits and of the H3K27 demethylase JMJD3 were recently shown not to be altered by the EBV or EBNA3 status in B cells [32]. Thus, PcG silencing of the *CXCL10/9* domain in the presence of EBNA3A cannot be explained by an increased availability of PRC2 or decreased expression of H3K27 demethylases.

**Figure 4:**
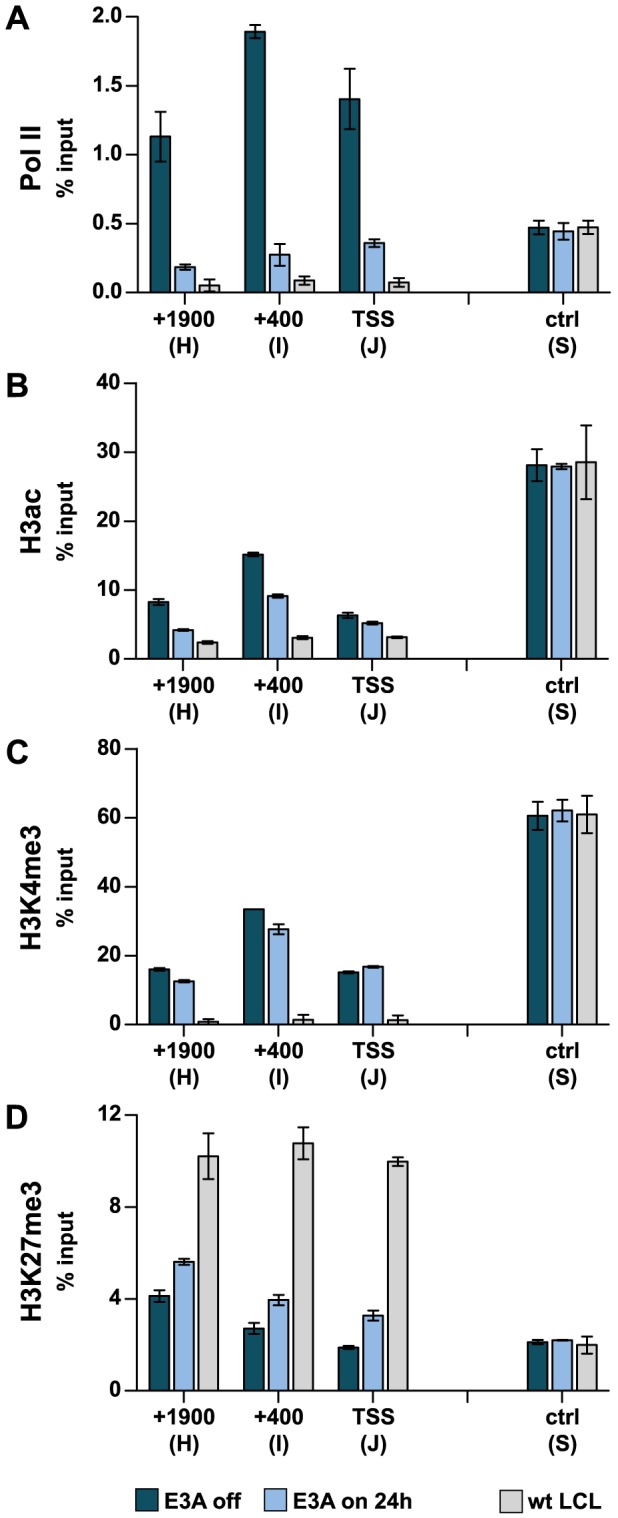
Transcriptional down-regulation precedes the gain of repressive H3K27me3 chromatin marks. ChIP analysis of ΔE3A-LCL^doxE3A^ cells showing the abundance of (A) Pol II, (B) H3ac, (C) H3K4me3, and (D) H3K27me3 across the *CXCL10* locus (primer pairs H-J, see Figure 3A) prior to and 24 h post EBNA3A induction with 100 ng/ml Dox. Primer pair S was used as a control. Bars were calculated and displayed as in Figure 3. ChIP analyses of a wt LCL were included for comparison.

Based on recent studies it could be suggested that EBNA3 proteins repress cellular genes by increasing H3K27 trimethylation [27], [29]–[33]. Measurements of *CXCL10* and *9* transcription in ΔE3A-LCL^doxE3A^ cells had shown that transcription of both genes is rapidly down-regulated within 24 h upon EBNA3A expression (Figure 2F). If H3K27 trimethylation of the *CXCL10* and *9* loci would be the decisive factor for repression, we would have expected that this modification arises with similar kinetics as the transcriptional shut-down. To challenge this assumption, the abundance of H3K27me3, H3K4me3, H3ac, and Pol II across the *CXCL10* locus was assessed by ChIP analyses prior to and 24 h post EBNA3A induction. In parallel with the diminished transcription of *CXCL10* described above (Figure 2F) a substantial decrease of Pol II abundance was detected (Figure 4A). In contrast, the increase of H3K27me3 marks was surprisingly small and accompanied by a subtle decrease of the activation marks H3K4me3 and H3ac (Figure 4B, C, D). Remarkably, this weak gain of H3K27me3 was seen again throughout the domain and restricted by domain borders (Figure S3). 48 h post EBNA3A induction H3K27me3 levels were further increased (Figure S4). However, while repression of transcription was rapidly executed, the levels of H3K27me3 upon short-term expression of EBNA3A stayed far below the level seen in wt LCLs.

In order to test if repression by EBNA3A follows similar kinetics at other target gene loci, we selected the *CDH1*, *GIMAP4* and *ADAMDEC1* genes for further analysis. *CDH1* (Cadherin 1/E-cadherin) is repressed by EBNA3A [24] but up-regulated by EBNA3C and -2 [25], [51]. *GIMAP4* (GTPase, IMAP family member 4) is one of several EBNA3A repressed genes located within the *GIMAP* gene cluster [24], which is directly targeted by EBNA2 and its cellular analogue NOTCH [19], [52]. *ADAMDEC1* (ADAM-like, decysin 1) can be up-regulated by EBNA2 [53] and, together with its genomic neighbor *ADAM28*, forms an EBNA3A and -3C co-repressed gene cluster that was already shown to be directly targeted by EBNA3C [29]. At each of these three gene loci transcriptional activity decreased already 24 h post EBNA3A induction (Figure S5A). 48 h post EBNA3A induction Pol II occupancy was reduced to levels that were similar to those observed in wt LCLs (Figure S5B). In contrast, a significant increase of H3K27me3 histone marks could again not be detected (Figure S5C). In summary, these data indicate that repression by EBNA3A is initiated by a loss of Pol II that precedes the gain of repressive PcG signatures at at least 4 independent gene loci.

### The PRC2 signature is established to wt levels on transcriptionally inactive genes and maintained by EBNA3A

**Figure 5:**
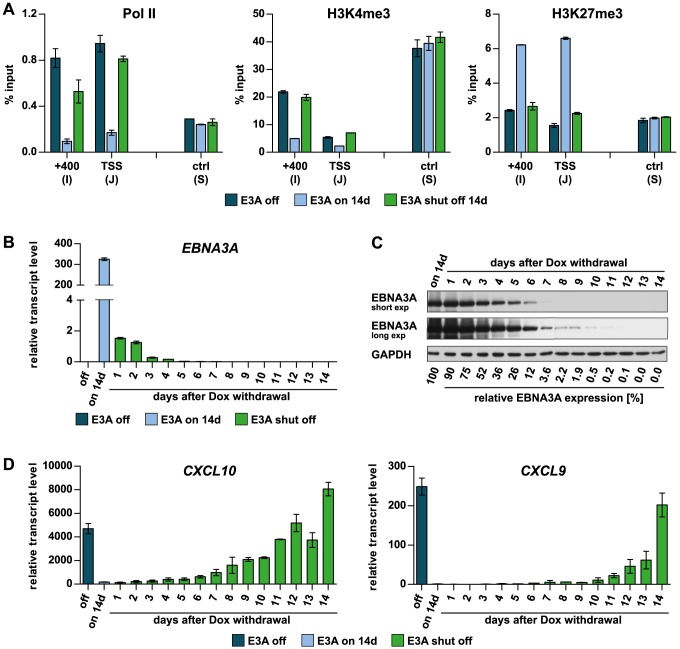
Maintenance of the Polycomb signature depends on EBNA3A. (A) ChIP analysis of ΔE3A-LCL^doxE3A^ cells showing the occupancy of Pol II, H3K4me3, and H3K27me3 at the *CXCL10* locus (primer pairs I and J, see Figure 3A) prior to, after 2 weeks of EBNA3A expression, and 2 weeks after EBNA3A shut-off. Results were calculated and displayed as in Figure 3. Primer pair S was included as a control. (B) qPCR quantification of *EBNA3A* transcripts in RNA extracts prepared at the indicated points in time. Results were normalized to 18S rRNA levels and are given as mean ± SD of triplicate qPCR reactions. (C) Western blot analysis of protein extracts using α-EBNA3A antibody. GAPDH detection was used as a loading control. Protein band intensities were quantified by densitometry. EBNA3A protein levels were normalized to GAPDH and are indicated as the percentage of EBNA3A protein remaining after Dox withdrawal relative to the expression level seen before Dox withdrawal. (D) qPCR analysis of *CXCL10* and *9* re-expression upon EBNA3A shut-off using the same RNA extracts as in panel (B). Results were normalized to 18S rRNA levels and are given as mean ± SD of triplicate qPCR reactions.

Next we asked whether H3K27me3 marks increase to wt levels during prolonged expression of EBNA3A and if repression at this “chromatin fixed” stage is reversible. To this end, we analyzed the abundance of H3K27me3, H3K4me3, and Pol II across the *CXCL10* locus prior to, after 2 weeks of EBNA3A expression in ΔE3A-LCL^doxE3A^ cells, and after subsequent EBNA3A shut-off for additional 2 weeks (Figure 5A). Following 2 weeks of EBNA3A expression, the abundance of H3K27me3 was substantially increased and almost reached the range detected in wt LCLs. Concomitantly, the activation mark H3K4me3 was found to be erased. When EBNA3A expression was subsequently shut-off by Dox withdrawal, transcripts of *EBNA3A* rapidly dropped within 24 h (Figure 5B). In contrast, the EBNA3A protein disappeared gradually within a period of 10–12 days post Dox withdrawal (Figure 5C). This kinetic is consistent with the finding that EBNA3A is remarkably stable and has a very slow turnover [54]. In accordance with the gradual decline of EBNA3A protein levels *CXCL10* and *9* transcription was gradually de-repressed (Figure 5D). Consistent with full re-expression of *CXCL10* and *9* the repressive H3K27me3 mark was found to be erased after 2 weeks of Dox withdrawal, while H3K4me3 levels were re-established to the initial range detected in ΔE3A-LCL^doxE3A^ cells prior to EBNA3A induction (Figure 5A). In summary, the data suggest that the increase of H3K27me3 upon EBNA3A induction up to levels observed in wt LCLs requires at least 14 days. Establishment and maintenance of the full PcG signature, however, clearly depends on the presence of EBNA3A, since it is fully reversed when EBNA3A is shut-off. The reversion of PcG silencing was also observed after 2 month of EBNA3A expression and subsequent EBNA3A shut-off (Figure S6). Hence, maintenance of repressive PcG signatures appears to depend on EBNA3A at any time.

### EBNA3A repression of *CXCL10* and *CXCL9* requires CBF1 and impairs IFNγ responsiveness

**Figure 6:**
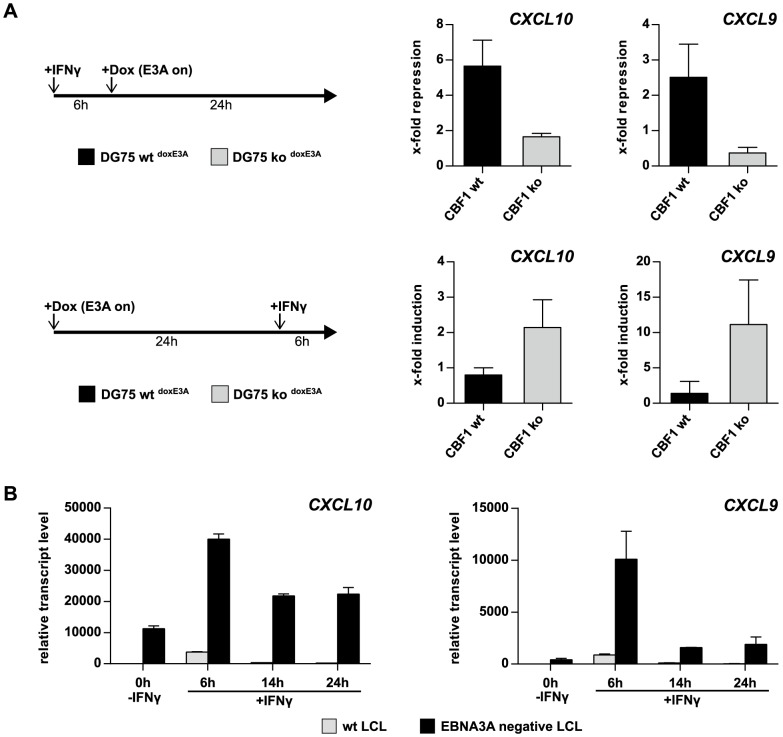
EBNA3A impairs *CXCL10* and *9* induction by IFNγ via a CBF1-dependent mechanism. (A) Analysis of *CXCL10* and *9* repression by EBNA3A in DG75 wt^doxE3A^ and DG75 ko^doxE3A^ cell lines. EBNA3A expression was induced with 100 ng/ml Dox for 24 h either post (upper panels) or prior to (lower panels) IFNγ treatment for 6 h. *CXCL10* and *9* transcripts were quantified by qPCR prior to and post IFNγ or Dox treatment and normalized to 18S rRNA levels. X-fold repression and induction values are given as mean ± SD of two biological replicates analyzed in triplicates. (B) qPCR quantification of *CXCL10* and *9* transcripts prior to and post IFNγ treatment of a wt and an EBNA3A negative LCL established from the same B cell donor. Transcript levels were normalized to 18S rRNA levels and are shown as mean ± SD of three independent experiments.

In the past we had generated a *CBF1* negative DG75 Burkitt's lymphoma cell line by targeted gene deletion [55]. To determine whether *CXCL10* and *9* repression by EBNA3A requires CBF1, we now stably transfected the isogenic DG75 B cell lines which either express or lack CBF1 with the episomal vector system that drives EBNA3A and NGFR expression after induction with Dox. The resulting cell lines were termed DG75 wt^doxE3A^ and DG75 ko^doxE3A^, respectively. Expression of NGFR and EBNA3A prior to and post Dox treatment was analyzed by flow cytometry and western blotting and was similar in both cell lines (Figure S7A, B). Transcripts of *CXCL10* and *9* were low but could be induced in both cell lines by IFNγ treatment in the absence of EBNA3A, reached a half-maximal expression after 6 h, and remained elevated for at least 30 h (Figure S7C). Importantly, induction of *CXCL10* and *9* was similar in DG75 wt and CBF1 knock out cells, indicating that CBF1 is not compulsory for induction by IFNγ. In order to test whether EBNA3A can reverse IFNγ-mediated induction, cells were treated with IFNγ for 6 h and subsequently induced for EBNA3A expression for 24 h. In DG75 wt^doxE3A^ cells expression of EBNA3A fully repressed the IFNγ driven expression of *CXCL10* and, albeit to a lesser extent, of *CXCL9*. In contrast, *CXCL10* and *9* repression was severely impaired when EBNA3A was expressed in IFNγ treated DG75 ko^doxE3A^cells (Figure 6A).

We next asked whether EBNA3A can even prevent IFNγ-mediated induction of the two genes by changing the experimental set-up. Now EBNA3A was induced for 24 h prior to IFNγ treatment. EBNA3A abolished IFNγ-mediated induction of *CXCL10* and *9* in CBF1 positive but not in CBF1 deficient cells (Figure 6A). In agreement with our previous findings, these results again highlight the presence of CBF1 as a prerequisite for EBNA3A's mode of action. Thus, our results substantiate for the first time that efficient repression of cellular genes by EBNA3A requires CBF1. Since the analyses were performed in EBV negative B cells, they also indicate that EBNA3A is sufficient to repress the two genes even in the absence of other latent EBV proteins.

To determine whether EBNA3A also impairs IFNγ responsiveness of *CXCL10* and *9* in EBV infected B cells, we analyzed wt and EBNA3A negative LCLs upon IFNγ treatment. In fact, IFNγ-mediated induction of *CXCL10* and *9* was strongly impaired in wt LCLs (Figure 6B). In contrast, IFNγ treatment of EBNA3A negative LCLs dramatically increased *CXCL10* and *9* transcript levels. In summary, repression by EBNA3A appears to be a dominant effect that even impairs the end points of CBF1 unrelated pathways like IFN signaling in EBV infected B cells.

### EBNA3A directly targets intergenic enhancers between *CXCL10* and *CXCL9* that are also bound by CBF1 and EBNA2

**Figure 7:**
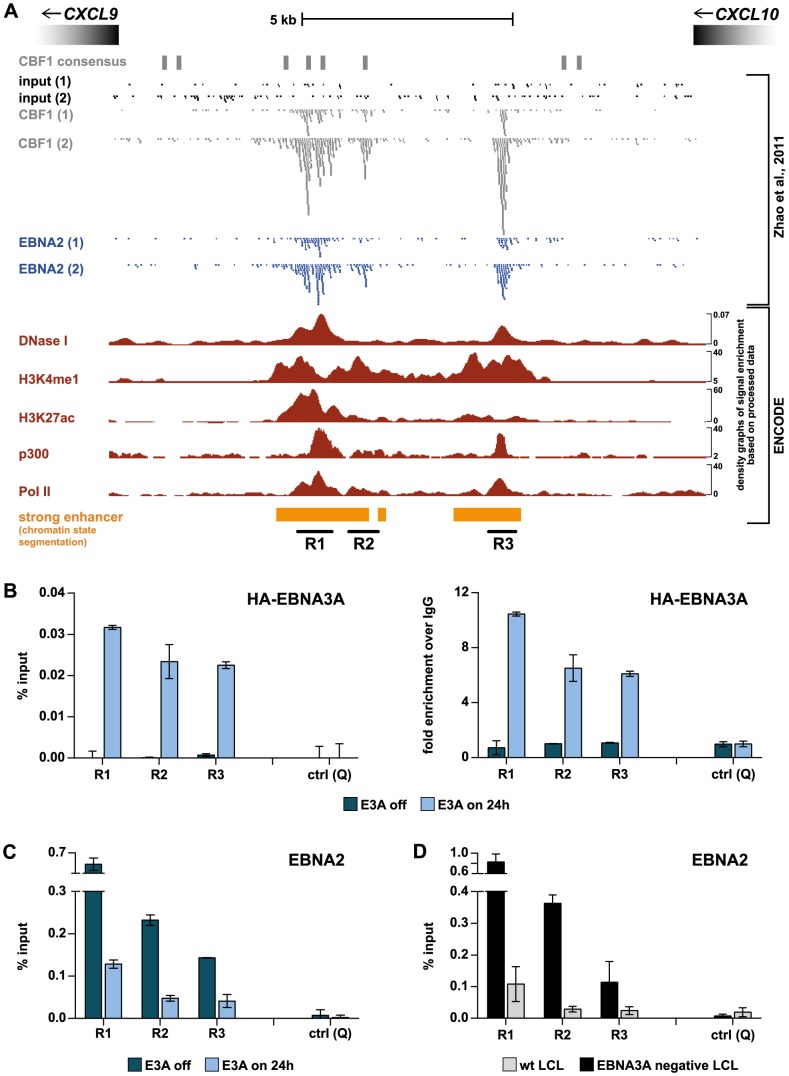
EBNA3A directly targets intergenic enhancers between *CXCL10* and *9* that are also bound by CBF1 and EBNA2. (A) Close-up of enhancer regions R1–R3 which are clustered within an intergenic 6 kb region located between *CXCL10* and *9*. R1–R3 are bound by CBF1 and EBNA2 in LCLs according to published ChIP-seq results [19], which are displayed as raw read data for EBNA2, CBF1, and input DNA duplicates. The depicted region was additionally analyzed for CBF1 consensus binding sites [100] and aligned with ENCODE DNase-seq data, ChIP-seq data for H3K4me1, H3K27ac, p300, and Pol II, as well as strong enhancer annotations revealed by chromatin state segmentation. All displayed ENCODE data were generated with wt LCLs (GM12878). Black lines demarcate region R1, R2, and R3. (B) ChIP analysis with α-HA antibody showing the binding of HA-tagged EBNA3A to regions R1–R3 24 h post HA-EBNA3A induction with 100 ng/ml Dox in ΔE3A-LCL^doxHA-E3A^ cells. Results were either calculated as described in Figure 3 (left panel) or displayed as fold enrichment of α-HA precipitated DNA over negative control IgG precipitation (right panel). Primer pair Q (see Figure 3A) shows neither CBF1 nor EBNA2 binding and was used as a negative control. (C) ChIP analysis of EBNA2 occupancy at regions R1–R3 prior to and 24 h post HA-EBNA3A induction with 100 ng/ml Dox in ΔE3A-LCL^doxHA-E3A^ cells. Results were calculated and displayed as in Figure 3. Primer pair Q was used as a negative control. (D) ChIP analysis of EBNA2 occupancy at regions R1–R3 in established wt and EBNA3A negative LCLs. Results are shown as mean ± SD of two independent experiments analyzed in duplicates. Primer pair Q was used as a negative control.

A genome-wide description of EBNA2 and CBF1 binding sites in EBV infected B cells was published recently [19]. By browsing these data sets we could identify a cluster of 3 EBNA2 and CBF1 bound regions (R1–R3) within an intergenic 6 kb region located between *CXCL10* and *CXCL9* (Figure 7A). Regions R1–R3 displayed elevated PRC2 and H3K27me3 occupancy in wt compared to EBNA3A negative LCLs and showed increasing H3K27me3 levels upon EBNA3A expression in ΔE3A-LCL^doxE3A^ cells (Figure S8). Remarkably, R1–R3 have already been annotated as strong enhancers in EBV infected B cells by chromatin state segmentation approaches [56], [57]. Regions R1–R3 carry chromatin signatures characteristic for enhancers including DNase hypersensitivity [58], association with p300 [59], [60], monomethylation of histone H3 at lysine 4 (H3K4me1) [59],[61],[62], acetylation of histone H3 at lysine 27 (H3K27ac) [63], [64], and association with Pol II [65]–[68]. In order to test if EBNA3A directly binds to enhancer regions R1–R3, we performed ChIP analyses. To date, there are no EBNA3A specific antibodies available, which can be used to detect EBNA3A on DNA by ChIP analysis that do not cross-react with other viral proteins. We thus generated an EBNA3A negative LCL that expresses HA-tagged EBNA3A upon Dox treatment (ΔE3A-LCL^doxHA-E3A^). Strikingly, regions R1, R2, and R3 were significantly enriched in HA-EBNA3A precipitates in Dox treated but not in untreated ΔE3A-LCL^doxHA-E3A^ cells (Figure 7B). The amount of HA-EBNA3A precipitated DNA was low when compared to the amount of input DNA but enriched several fold over the levels of DNA precipitated with a negative control antibody (Figure 7B). HA-EBNA3A binding was not observed at a negative control locus that was located within the PcG-controlled *CXCL10/9* domain and is neither bound by CBF1 nor EBNA2 [19].

Since EBNA2 and EBNA3A are invariably co-expressed in EBV infected B cells, bind CBF1, and show competitive activities in reporter gene assays (see introduction), it has been postulated that the two viral factors are antagonists. In order to test if EBNA3A and EBNA2 compete for CBF1 binding sites, we analyzed EBNA2 occupancy at R1, R2, and R3 in the absence and presence of EBNA3A. Strikingly, EBNA2 occupancy was drastically elevated at regions R1–R3 in the absence of HA-EBNA3A when compared to ChIP results obtained 24 h after HA-EBNA3A induction in ΔE3A-LCL^doxHA-E3A^ cells (Figure 7C). The EBNA2 ChIP data obtained prior and post HA-EBNA3A expression suggested that EBNA2 binding to regions R1–R3 should be stronger in EBNA3A deficient LCLs relative to wt LCLs. Indeed, EBNA2 occupancy was significantly elevated at R1–R3 in EBNA3A negative LCLs when compared to wt LCLs (Figure 7D). These results provide the first direct evidence that EBNA3A and EBNA2 can compete for access to CBF1 at identical sites of the cellular genome.

### EBNA3A binding to intergenic enhancers causes a switch in enhancer activity

**Figure 8:**
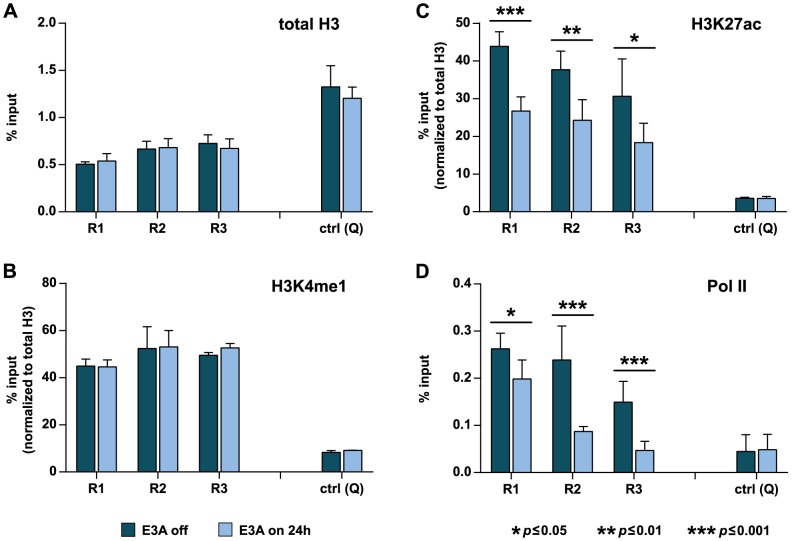
EBNA3A binding to intergenic enhancers reduces enhancer activity. ChIP analysis of (A) histone H3, (B) H3K4me1, (C) H3K27ac, and (D) Pol II occupancy at regions R1–R3 prior to and 24 h post HA-EBNA3A induction with 100 ng/ml Dox in ΔE3A-LCL^doxHA-E3A^ cells. Data are shown as mean ± SD of three independent experiments analyzed in duplicates. Results for H3K4me1 and H3K27ac were normalized to total histone H3 levels to account for the low nucleosomal occupancy at regions R1–R3. Asterisks indicate the *p*-value as determined by Student's *t*-test. Primer pair Q was used as a negative control.

The reciprocal binding patterns of the transactivator EBNA2 and the repressor EBNA3A at intergenic enhancers suggested a switch in enhancer activity. Low nucleosome occupancy and H3K4me1 modifications mark enhancers of transcriptionally active as well as PcG-repressed genes [41]. Indeed, histone H3 occupancy and the abundance of H3K4me1 at regions R1–R3 did not change 24 h post HA-EBNA3A induction (Figure 8A, B). In contrast, marks that define active enhancers like H3K27ac and Pol II occupancy [63]–[67], [69] demonstrated a significant shift in enhancer activity. H3K27ac abundance within the R1–R3 cluster was reduced 24 h post HA-EBNA3A induction (Figure 8C). In addition, Pol II occupancy was significantly diminished at regions R2 and R3, while loss of Poll II at R1 was less pronounced (Figure 8D). The individual regions might thus differ in their contribution to control adjacent genes in a way that remains to be studied. In summary, our findings strongly suggest that EBNA3A primarily acts by reducing the state of enhancer activity, which is otherwise maintained by the transactivator EBNA2 in a CBF1-dependent manner.

## Discussion

The CXCL10 and 9 chemokines and their receptors on NK and T cells are critical weapons of the infected host to control herpesvirus infections [70]–[72]. In EBV infected B cells *CXCL10* and *9* expression can be triggered by IFNs and TNFα [73], which are equally expressed in wt as well as EBNA3A negative LCLs [24]. Importantly, *CXCL10* expression is fine-tuned by multiple mechanisms in EBV infected B cells, suggesting an important role for this chemokine in the viral life cycle. The latent proteins EBNA2 and -3B can enhance while EBNA3A and -3C can repress *CXCL10* expression [24], [29], [74], [75].

Here, we decided to analyze repression of the genes *CXCL10* and *9* by EBNA3A, since they combine two characteristic features of EBNA3A repressed targets. Like the majority of EBNA3A repressed genes they carry a PcG signature in the repressed state and they form a pair of co-regulated genes (Figure 1, 2A). Importantly, our analysis also revealed that genes, which carry a PcG signature in EBV infected B cells, can also carry this signature in multiple EBV negative tissues of different origin (Figure 1B, 1C), indicating that EBNA3A might use intrinsic properties of the respective gene loci commonly employed by the cell to recruit PcG proteins.

Using the *CXCL10* and *9* genes as a model, we found that EBNA3A rapidly reduces transcription of both genes in a CBF1-dependent manner and in the following initiates the gain of PRC2-catalyzed H3K27me3 marks, which gradually increase within the following two weeks in cell culture. Upon EBNA3A withdrawal, H3K27me3 marks were erased within two weeks. Rapid *CXCL10* and *9* shut-down and gradual initiation of PcG silencing coincided with EBNA3A's binding to intergenic enhancers located between the two genes. Simultaneously, EBNA2 was displaced and enhancer activity was diminished. Thus, we identified the first direct and CBF1-dependent target genes of EBNA3A, demonstrated reciprocal binding patterns of EBNA3A and -2 at identical genomic enhancer sites, and revealed that these genomic enhancers are critical hubs that can integrate co-regulation of neighboring genes with re-programming of chromatin states.

### Transcriptional down-regulation of *CXCL10* and *9* precedes the gain of repressive PcG signatures

EBNA3C and -3A share a homologous N-terminal domain which mediates CBF1 binding and both proteins score as repressors when tethered to DNA by GAL4. In addition, they physically interact [32], [76] and share a significant set of co-regulated host target genes which may carry PcG signatures in the repressed state [25], [27], [29]–[33], [77], [78]. Since EBNA3A and -3C share so many features, it could be assumed that they control gene expression by similar mechanisms.

Recently, repression of the *p16* tumor suppressor gene by a conditional EBNA3C was studied in time course experiments. This study used a hydroxytamoxifen dependent EBNA3C protein fused to a modified estrogen receptor (EBNA3CHT). EBNA3CHT induced a slow decrease of *p16* transcript levels and simultaneous increase of H3K27me3 marks, suggesting that transcriptional repression by EBNA3C is caused by H3K27 trimethylation [33]. Our study confirmed a slow increase in H3K27me3 occupancy across the *CXCL10* and *9* genes in the presence of EBNA3A. However, using a Dox-based EBNA3A conditional LCL, we show here that EBNA3A expression causes a rapid transcriptional shut-down of *CXCL10* and *9* that coincides with reduced Pol II recruitment to promoter and enhancer sites (Figure 2F, 4A, 8D) and precedes the gain of repressive H3K27me3 signatures that rather constitute a consequence but not the cause of transcriptional shut-down (Figure 4D, S3, S4).

A direct comparison of our results with previously published studies is hampered by the fact that repression of (i) two different gene loci by (ii) two distinct viral proteins was analyzed in (iii) two distinct conditional systems. In order to directly compare EBNA3A and -3C, it would have been interesting to study repression of *p16* by EBNA3A. Unfortunately, *p16* expression can get lost spontaneously in cell culture in our EBNA3A negative LCLs and thus cannot be studied systematically in EBNA3A conditional LCLs. We thus switched to three additional target genes of EBNA3A: *CDH1*, *GIMAP4*, and *ADAMDEC1*. Like *p16*, *ADAMDEC1* is repressed by both, EBNA3A and EBNA3C. Remarkably, we again observed a rapid reduction of transcription followed by a marginal gain of H3K27me3 histone modifications at all three EBNA3A target genes, suggesting that this mode of action is a general feature of EBNA3A (Figure S5).

We feel that the use of different conditional systems might explain the disparate results of our study and previously published studies. In order to study *p16* repression by EBNA3CHT, the viral protein was inactivated for several weeks in culture. This causes a proliferative arrest of EBNA3CHT LCLs. Repression of *p16* by EBNA3CHT was subsequently studied after re-induction of the viral protein in growth arrested cultures [33]. A subpopulation of these cells might be driven into a state of irreversible arrest similar to senescence [33]. These would be refractory to further EBNA3CHT signals and might have hampered the detection of a rapid transcriptional shut-down. An alternative explanation for our disparate results might be the high expression levels of EBNA3A in our Dox-based system. Overexpression of EBNA3A might have forced the rapid transcriptional shut-down of *CXCL10* and *CXCL9*, and of the genes *CDH1*, *GIMAP4* and *ADAMDEC1*. This might have enabled us to observe that transcriptional repression by EBNA3A follows faster kinetics than the delayed gain of repressive H3K27me3 histone marks.

However, observations made on one particular EBNA3 protein might even not be transferable to other EBNA3 proteins. EBNA3A and -3C have unique biological functions and cannot complement each other [79], [80]. Both viral proteins might also act by distinct mechanisms on the same gene.

### Maintenance of PcG silencing requires EBNA3A

While PcG proteins are unlikely to be involved in the initial transcriptional shut-down of *CXCL10* and *9* by EBNA3A, the establishment and maintenance of the PcG signature was strictly EBNA3A-dependent. When EBNA3A expression was discontinued, H3K27me3 marks were lost and re-expression of *CXCL10* and *9* was permitted (Figure 5, S6). Thus, PcG silencing of *CXCL10* and *9* is not taken over by cellular systems and perpetuated in a “hit and run mechanism”. Instead, maintenance of PcG silencing appears to depend on EBNA3A at any time. Hence, like PcG silencing of *BIM* and *p16* by EBNA3C [32], [33], PcG silencing of *CXCL10* and *9* by EBNA3A is reversible. Since both, EBNA3A and -3C, can easily switch PcG patterns on cellular genes, they appear to mimic a cellular stimulus or protein that impinges on a decision-making level of the PcG recruitment system.

### Molecular competition of EBNA3A and EBNA2 on remote enhancers

This study demonstrated that EBNA3A requires CBF1 for repression of *CXCL10* and *9* (Figure 6A) and directly binds to intergenic enhancers located between both genes, which have been previously shown to recruit CBF1 and EBNA2 in LCLs (Figure 7A, B) [19]. These findings suggest that, like EBNA2, EBNA3A is targeted to DNA by CBF1. EBNA2 and -3A were repeatedly suggested to be competitive antagonists based on reporter gene assay data [14]–[18]. Actually, EBNA2 transactivation of *CXCL10* was previously reported in EBNA3A negative B cell lines [29], [74]. We now show that EBNA2 and -3A establish reciprocal binding patterns at intergenic enhancers within the *CXCL10/9* domain (Figure 7). Enhancer binding patterns clearly correlated with distinct enhancer activity and expression levels of adjacent genes (Figure 2, 7, 8). Thus, the viral proteins appear to compete for access to identical genomic sites bound by CBF1, the common DNA adaptor.

During the course of EBV infection, EBNA2 expression precedes expression of EBNA3A. Thus, strong expression of *CXCL10* and *9* might only be licensed in the initial phase due to the unrestricted positive effect of EBNA2 on the intergenic enhancer regions within the *CXCL10/9* domain. In B cells co-expressing EBNA2 and -3A, the primary activity of EBNA3A might be to displace the EBNA2 transactivator. However, as shown here, EBNA3A also down-regulates and even prevents IFNγ-mediated induction of *CXCL10* and *9* in EBNA2 negative DG75 wt B cell lines (Figure 6A), indicating that EBNA3A uses its intrinsic repressor activity to silence gene expression induced by cellular signaling pathways. Since EBNA3A abolished IFNγ-mediated induction of *CXCL10* and *9* in CBF1 positive but not in CBF1 deficient DG75 cells (Figure 6A), enhancer repression by EBNA3A appears to be the dominant process that even impairs *CXCL10* and *9* induction by CBF1-independent pathways like IFN signaling, which act upstream.

Interestingly, we found EBNA2 and CBF1 to bind to several distal enhancers located within co-repressed EBNA3A target gene clusters, when we combined EBNA2 and CBF1 binding sites [19], putative enhancer annotations [56], [57], and our panel of EBNA3A repressed genes [24] (data not shown). Examples include the genomic neighbors *ADAMDEC1* and *ADAM28*, as well as the genes *GIMAP4*, *GIMAP5*, and *GIMAP6* located within the *GIMAP* gene cluster. We speculate that transcriptional repression of these genes and subsequent epigenetic changes might also be caused by EBNA3A's binding to and inactivation of EBNA2/CBF1 occupied enhancers.

### A 2-step model for initiation and maintenance of *CXCL10/9* repression by EBNA3A

**Figure 9:**
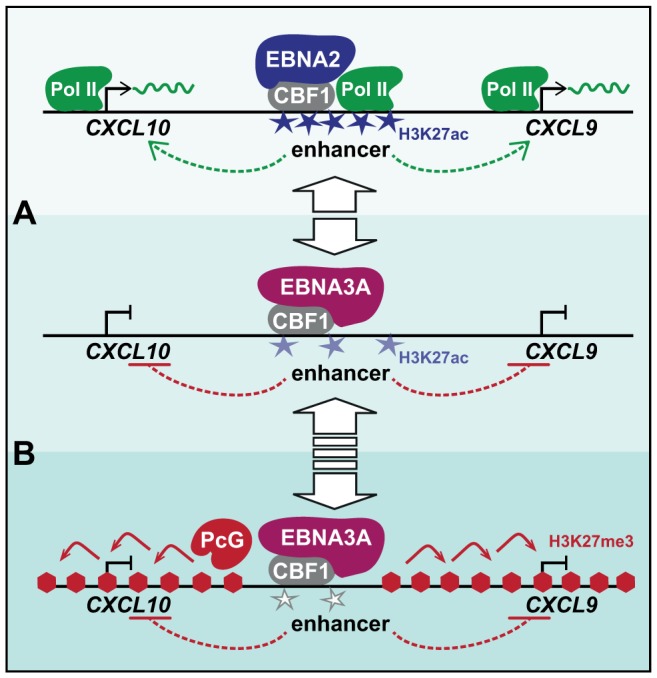
A 2-step model for EBNA3A's mode of action. (A) EBNA3A displaces the transactivator EBNA2 from CBF1 occupied intergenic enhancers. Reduction of EBNA2 triggered enhancer activity by EBNA3A binding causes a rapid transcriptional shut-down of adjacent *CXCL10* and *9* genes. In the absence of EBNA2, however, EBNA3A acts by its intrinsic repressor activity, rendering *CXCL10* and *9* refractory to IFNγ-mediated induction. (B) The transcriptionally repressed state of *CXCL10* and *9* is subsequently fixed on the chromatin level by PcG proteins. PRC2-catalyzed H3K27me3 marks spread in a domain-wide fashion, potentially starting from remote enhancers. The gain of H3K27me3 levels to full range is a slow process that requires a time period of at least 14 days. When EBNA3A expression is discontinued, PcG repression is reversed and re-expression of distal genes is permitted (blue stars: H3K27ac; red hexagons: H3K27me3).

Based on our present findings, we suggest a 2-step model for gene repression by EBNA3A that is depicted in Figure 9. We propose that the rapid transcriptional shut-down of *CXCL10* and *9* is caused by EBNA3A's binding to and inactivation of intergenic enhancers (Figure 9A). EBNA3A interfered with EBNA2 and Pol II recruitment as well as H3K27 acetylation. Pol II binding at active enhancers is discussed to facilitate Pol II loading onto promoters by physical enhancer-promoter interaction [20]. Since enhancers can be shared by adjacent genes [81], [82], a reduced enhancer recruitment of Pol II can affect the expression of multiple genes. This might explain the down-regulation of multiple co-repressed gene clusters by EBNA3A. In addition, recent studies reported that enhancers are transcribed and give rise to RNAs [66], [67], [83]. The functionality of enhancer transcription might be conveyed by the active transcription process or by enhancer derived non-coding RNAs which exert enhancer functions directly or by recruitment of cellular chromatin modifiers [84]. In summary, we suggest that the reduced recruitment of Pol II to enhancers is the initial cause for rapid transcriptional shut-down of adjacent *CXCL10* and *9* genes (Figure 9A).

In a second delayed step PcG signatures are established across the *CXCL10/9* domain (Figure 9B). The molecular mechanism of domain-wide PcG silencing upon EBNA3A's binding to and inactivation of intergenic enhancers remains to be established. Polycomb eviction was described as an enhancer function recently [42]. In addition, enhancers provide a binding platform for master transcription factors that initiate epigenetic changes in associated promoters and reprogramming of PcG-repressed genes [41]. Enhancers can also recruit chromatin modifying enzymes that then spread domain-wide [20], [85], [86]. Since PcG proteins control multiple NOTCH targets in flies and mammals [87], [88], CBF1 or components of the associated co-repressor complex might convey PcG recruitment in the absence of NOTCH/EBNA2 signals. In addition, we do not want to exclude that EBNA3A recruits PcG complexes by physical interaction. However, it has to be considered that PcG silencing can constitute the default state of genes if not actively counteracted by antagonizing chromatin modifiers or an active transcription process through recruiting DNA elements [89]–[93]. Since H3K27ac, H3K4me3, and transcription associated H3K36 methylation interferes with PcG silencing [90], [94], [95], and enhancer Pol II occupancy can govern histone modifications in genes and *cis*-regulatory regions [96], we speculate that enhancer inactivation by EBNA3A might be sufficient to trigger PcG silencing across the *CXCL10/9* domain.

At this point of our study, we are confident that PcG silencing spreads across the *CXCL10/9* domain subsequent to the transcriptional shut-down of target genes and as a consequence of EBNA3A's binding to and inactivation of remote enhancers.

## Materials and Methods

### Bioinformatics

Bioinformatics data processing and ENCODE data sets are described in the supporting information section (Text S1).

### Plasmids

The episomal vector system pRTS-1 [48] carries a bidirectional Dox-inducible promoter which drives the equivalent expression of NGFR and a gene of interest simultaneously. EBV type I EBNA3A (B95.8 strain) was inserted into pRTS-1 using standard cloning procedures. A Gateway-compatible pRTS-1 derivative was used to add in-frame an HA-tag to the N-terminus of EBNA3A upon recombination of expression clones by Clonase enzymes (Invitrogen).

### Cell lines

LCLs established by infection of primary human B cells with EBVwt, EBV-E3AmtA or EBV-E3AmtB [24], and the cell lines DG75 wt [97], and DG75 *CBF1* knock out [55] were cultivated as described [24]. IFNγ treatment was accomplished by cultivation with human IFNγ (Miltenyi Biotech) at 3000 U/ml. The cell lines ΔE3A-LCL^doxE3A^ and ΔE3A-LCL^doxHA-E3A^ were established by transfection of the EBNA3A negative LCL D2 E3AmtB 3 [24] with respective pRTS-1 derivatives. The cell lines DG75 wt^doxE3A^ and DG75 ko^doxE3A^ were established by transfection of the cell lines DG75 wt and DG75 *CBF1* knock out with respective pRTS-1 derivatives. Stable cell lines were selected with puromycin as described in the supporting information section (Text S2).

### Flow cytometry

Cells were stained with mouse α-human NGFR antibody (HB8737, ATCC) or an isotype control (mouse α-GST antibody, 2C8, E. Kremmer) and with Cy5-coupled goat α-mouse antibody (Dianova) and analyzed using a FACSCalibur system (BD Biosciences) and CellQuest Pro software (BD Biosciences).

### Western blot analysis

Western blots were probed with the following primary antibodies: α-EBNA3A (E3AN4A5, E. Kremmer), α-CBF1 (RBP-J 7A11, E. Kremmer), and α-GAPDH (Millipore). HRP-coupled secondary antibodies (Santa Cruz Biotechnology) and an ECL kit (GE Healthcare) were used for visualization. For quantification of protein levels, exposed films were scanned in transmission mode and protein band intensities were determined by densitometry using *ImageJ* software (http://rsbweb.nih.gov/ij/) [98]. Different exposure times were analyzed for quantification in Figure 5C and the protein extract at day 6 after Dox withdrawal was used as a reference value between films.

### 4sU labeling of nascent RNA, RNA preparation and cDNA synthesis

Nascent RNA was metabolically labeled by cultivating cells in cell culture media supplemented with 100 µM 4sU (Sigma-Aldrich) for 1 h. Total RNA preparation and purification of 4sU labeled nascent RNA was performed as described [50]. Otherwise, RNA was prepared using RNeasy Mini Kit (Qiagen), treated with RNase-Free DNase Set (Qiagen) and reverse transcribed using High Capacity cDNA Reverse Transcription Kit (Applied Biosystems). Transcript levels were quantified by qPCR as described below using primers listed in Table S3.

### Chromatin immunoprecipitation

ChIP analyses were performed at least 3 times as described [99] with minor modifications described in the supporting information section (Text S3). Antibodies used for ChIP are listed in Table S4. For quantification of DNA in input samples and after IP with specific antibodies and non-specific isotype controls qPCR was performed using primers given in Table S5.

### Quantitative PCR

qPCR was performed using LightCycler 480 SYBR Green I Master (Roche) on a LightCycler 480 II instrument (Roche) according to the manufacturer's protocol. Cycling conditions were 10 min at 95°C and 45 cycles of 3 sec at 95°C, 10 sec at 60–63°C (see Tables S3 and S5), and 20 sec at 72°C on a 96-well thermal block. PCR products were analyzed by melting curve analysis and tested for correct size by gel electrophoresis. Primers used for quantification are listed in Tables S3 and S5. To correct qPCR data for differences in PCR efficiencies, a standard curve was generated for each primer pair by using serial dilutions of PCR products or sheared chromatin as templates for amplification and plotting the Cp values against the known dilutions. To determine the absolute number of transcripts present in a sample, a standard curve of 5 dilutions was generated using known amounts of PCR products containing the target sequence. PCR products were derived from cDNA templates, purified by gel extraction (Qiagen), and diluted at predetermined concentrations (5×10^7^, 5×10^5^, 5×10^3^, 5×10^1^, 5×10^−1^ particles per µl) using MS2 RNA (Roche) as a carrier. Absolute quantification of transcripts was performed using 1/50 of the cDNA prepared from 1 µg RNA as a template and was based on the standard samples of known concentration and the respective PCR efficiency for each primer pair. Absolute transcript numbers were normalized to 18S rRNA levels and designated “relative transcript level” accordingly. The relative transcript numbers were adjusted to the range of absolute transcript numbers by applying a multiplication factor. Thus, the relative transcript levels reflect the approximate amount of transcripts detected in 1/50 of the cDNA preparation.

### Accession numbers [official gene symbol]

ADAMDEC1: 27299; ADAM28: 10863; ART3: 419; BIM [BCL2L11]: 10018; CBF1 [RBPJ]: 3516; CDH1: 999; CTBP1: 1487; CTBP2: 1488; CXCL9: 4283; CXCL10: 3627; CXCL11: 6373; EBNA-2: 3783761; EBNA-3A: 3783762; EBNA-3B/EBNA-3C: 3783763; EZH2: 2146; GAPDH: 2597; GIMAP4: 55303; GIMAP5: 55340; GIMAP6: 474344; IFNγ [IFNG]: 3458; JMJD3 [KDM6B]: 23135; MYC: 4609; NGFR: 4804; NOTCH1: 4851; NOTCH2: 4853; NUP54: 53371; p16 [CDKN2A]: 1029; p300 [EP300]: 2033; SDAD1: 55153; SUZ12: 23512; TNFα [TNF]: 7124; TOX: 9760; UTX [KDM6A]: 7403

## Supporting Information

Figure S1 **Characterization of the ΔE3A-LCL^doxE3A^ cell line.** Characterization of the EBNA3A conditional LCL established by stable transfection of an EBNA3A negative LCL (D2 E3AmtB 3) [24] with an episomal vector system [48] that facilitates Dox-inducible simultaneous expression of EBNA3A and NGFR. (A) Western blot analysis of potential background EBNA3A expression in the absence of Dox. Protein extracts were prepared over a period of 3 weeks. Protein extracts of a wt LCL (D2 wt 1) [24] derived from the same donor served as a positive control. GAPDH immunodetection was used as loading control. (B) Western blot analysis of EBNA3A protein levels prior to and 72 h post treatment with increasing amounts of Dox. Protein extracts of a corresponding wt LCL (D2 wt 1) served as a positive control. GAPDH immunodetection was used as loading control. (C) qPCR quantification of *EBNA3A* transcripts prior to and 72 h post treatment with increasing amounts of Dox. Transcript levels of *EBNA3A* were normalized to 18S rRNA levels.
(EPS)

Figure S2 **PcG silencing of the** ***CXCL10/9*** **domain in the presence of EBNA3A is not restricted to a specific B cell donor.** ChIP analysis of (A) Pol II, (B) H3ac, (C) H3K4me3, and (D) H3K27me3 abundance across the *CXCL10* and *9* encompassing domain in wt and EBNA3A negative LCLs derived from a different B cell donor (D6 wt and D6 E3AmtA). Results were calculated as described in Figure 3. The location of primer pairs is displayed in Figure 3A.
(EPS)

Figure S3 **The modest increase of H3K27me3 marks 24 h post EBNA3A induction is observed in a domain-wide fashion.** ChIP analysis of (A) H3K27me3 and (B) H3K4me3 abundance across the *CXCL10/9* domain prior to and 24 h post EBNA3A induction with 100 ng/ml Dox in ΔE3A-LCL^doxE3A^ cells. Bar height was calculated as described in Figure 3. The location of primer pairs is displayed in Figure 3A. ChIP results of a wt LCL were included for comparison.
(EPS)

Figure S4 **H3K27me3 marks further increase 48 h post EBNA3A induction.** ChIP analysis of (A) H3K27me3 and (B) H3K4me3 abundance across the *CXCL10* locus prior to and 24 or 48 h post EBNA3A induction with 100 ng/ml Dox in ΔE3A-LCL^doxE3A^ cells. Bar height was calculated as described in Figure 3. The location of primer pairs is displayed in Figure 3A. ChIP results of a wt LCL were included for comparison.
(EPS)

Figure S5 **Transcriptional down-regulation of the EBNA3A repressed target genes** ***CDH1*** **,** ***GIMAP4*** **, and** ***ADAMDEC1*** **precedes the gain of repressive H3K27me3 chromatin marks.** (A) EBNA3A induction in ΔE3A-LCL^doxE3A^ cells rapidly reduces *de novo* transcription of *CDH1*, *GIMAP4*, and *ADAMDEC1*. Nascent RNA was prepared prior to and after 24 or 48 h of EBNA3A expression as described in Figure 2E, F. Nascent *CDH1*, *GIMAP4*, and *ADAMDEC1* transcripts were quantified by qPCR, normalized to nascent 18S rRNA levels, and are given as mean ± SD of two experiments analyzed in triplicates. (B, C) ChIP analysis of (B) Pol II and (C) H3K27me3 occupancy at the TSS of *CDH1*, *GIMAP4*, and *ADAMDEC1* prior to and 48 h post EBNA3A induction with 100 ng/ml Dox in ΔE3A-LCL^doxE3A^ cells. Results were calculated as described in Figure 3 and are shown as mean ± range of two experiments. ChIP results of a wt LCL were included for comparison.
(EPS)

Figure S6 **PcG silencing remains reversible even after 2 month of EBNA3A expression in conditional LCLs.** (A) ChIP analysis of H3K27me3 occupancy at the *CXCL10* locus (primer pair I, see Figure 3A) prior to and after 8 weeks of EBNA3A expression in ΔE3A-LCL^doxE3A^ cells. Results were calculated as described in Figure 3 and are shown as mean ± SD of triplicate qPCR reactions. Primer pair S was used as a control. ChIP results of a wt LCL were included for comparison and demonstrate the gain of H3K27me3 marks to full range in ΔE3A-LCL^doxE3A^ cells. (B) Analysis of potential *CXCL10* re-expression after 8 weeks of EBNA3A expression and subsequent shut-off by Dox withdrawal. Transcript levels were quantified by qPCR and normalized to 18S rRNA levels.
(EPS)

Figure S7 **The DG75 wt^doxE3A^ and DG75 ko^doxE3A^ cell lines show similar IFNγ-mediated induction of** ***CXCL10*** **and** ***CXCL9*** **in the absence of EBNA3A.** (A) Flow cytometric analysis of NGFR expression in DG75 wt^doxE3A^ and DG75 ko^doxE3A^ cell lines prior to and 24 h post treatment with 100 ng/ml Dox. Staining of cells with isotype-matched nonspecific antibodies served as a negative control. (B) Western blot analysis for CBF1 and EBNA3A protein levels prior to and post treatment of DG75 wt^doxE3A^ and DG75 ko^doxE3A^ cell lines with 100 ng/ml Dox. The parental DG75 wt cell line and a wt LCL served as a control. GAPDH immunodetection was used as loading control. (C) Transcript quantification of *CXCL10* and *9* in DG75 wt^doxE3A^ and DG75 ko^doxE3A^ cell lines prior to and after 6, 24 or 30 h of IFNγ treatment. Transcript levels were quantified by qPCR and normalized to 18S rRNA levels. X-fold induction values for *CXCL10* and *9* are shown as mean ± SD of three independent experiments.
(EPS)

Figure S8 **Intergenic enhancer regions R1–R3 display elevated PRC2 and H3K27me3 occupancy in wt compared to EBNA3A negative LCLs and increasing H3K27me3 levels upon EBNA3A expression in ΔE3A-LCL^doxE3A^ cells.** (A) ChIP analysis of H3K27me3, SUZ12 and EZH2 occupancy at enhancer regions R1, R2, and R3 in wt and EBNA3A negative LCLs. Results were obtained from the same experiments shown in Figure 3E–G and were embedded into these data sets. (B) ChIP analysis of ΔE3A-LCL^doxE3A^ cells showing the abundance of H3K27me3 at enhancer regions R1, R2, and R3 prior to and 48 h post EBNA3A induction with 100 ng/ml Dox. Results were calculated as described in Figure 3 and are shown as mean ± SD of triplicate qPCR reactions. ChIP results of a wt LCL were included for comparison.
(EPS)

Table S1 **Average number of transcripts of** ***CXCL9*** **,** ***CXCL10*** **,** ***CXCL11*** **, and** ***ART3*** **in 5×10^4^ cells determined in triplicates for five independent wt and EBNA3A negative LCLs established from five unrelated B cell donors.**
(DOCX)

Table S2 **Expression levels of PRC2 subunits and H3K27 demethylases in wt and EBNA3A negative LCLs according to gene expression profiling data described previously.**
(DOCX)

Table S3 **Primers used for quantification of transcripts by qPCR.**
(DOCX)

Table S4 **Antibodies used for chromatin immunoprecipitation.**
(DOCX)

Table S5 **Primers used for qPCR quantification of DNA recovered in ChIP experiments.**
(DOCX)

Text S1 **Bioinformatics analysis and ENCODE data sets.**
(DOCX)

Text S2 **Establishment of EBNA3A conditional B cell lines.**
(DOCX)

Text S3 **Chromatin immunoprecipitation and analysis of recovered DNA.**
(DOCX)
