# Supplementary material for: Inactivation of Intergenic Enhancers by EBNA3A Initiates and Maintains Polycomb Signatures across a Chromatin Domain Encoding CXCL10 and CXCL9
Source: PLoS Pathog. 2013 Sep 19;9(9):e1003638. doi: 10.1371/journal.ppat.1003638 (PMC3777872; doi:10.1371/journal.ppat.1003638)
Supplement: Table S1 — Average number of transcripts of CXCL9 , CXCL10 , CXCL11 , and ART3 in 5×10^4^ cells determined in triplicates for five independent wt and EBNA3A negative LCLs established from five unrelated B cell donors. (DOCX) [file ppat.1003638.s009.docx]

**Table S1. Average number of transcripts of *CXCL9*, *CXCL10*, *CXCL11,* and *ART3* in 5x10^4^ cells determined in triplicates for five independent wt and EBNA3A negative LCLs established from five unrelated B cell donors. ^1^**

| **B cell donor** | **Virus type used for infection** | ***CXCL9*** | ***CXCL10*** | ***CXCL11*** | ***ART3*** |
| --- | --- | --- | --- | --- | --- |
| **D1** | EBV wt | 383.5 | 11784.0 | 64.7 | 0.0 |
|  | EBV E3AmtA | 22288.0 | 378480.0 | 133.0 | 0.1 |
| **D2** | EBV wt | 41.6 | 139.3 | 0.0 | 0.0 |
|  | EBV E3AmtB | 3775.3 | 450333.3 | 255.7 | 0.1 |
| **D3** | EBV wt | 24.0 | 4061.3 | 0.0 | 0.0 |
|  | EBV E3AmtB | 8506.0 | 190413.3 | 67.4 | 0.0 |
| **D4** | EBV wt | 4.8 | 167.2 | 7.9 | 0.0 |
|  | EBV E3AmtA | 6881.3 | 58785.3 | 77.8 | 0.0 |
| **D5** | EBV wt | 154.5 | 6161.3 | 41.7 | 0.0 |
|  | EBV E3AmtA | 144066.7 | 383866.7 | 109.7 | 0.1 |

^1^ RNA preparations from 1x10^7^ cells yielded 200 µg RNA on average. cDNA was prepared from 1 µg RNA (corresponding to 5x10^4^ cells) in triplicates. A total of 1/50 of the cDNA was analyzed by qPCR. Absolute quantification was performed based on standard samples of known particle numbers and the respective PCR efficiency for each primer pair (see materials and methods section). The absolute number of transcripts detected in a sample was multiplied by 50 to calculate the number of transcripts present in 5x10^4^ cells.
