# Supplementary material for: Inactivation of Intergenic Enhancers by EBNA3A Initiates and Maintains Polycomb Signatures across a Chromatin Domain Encoding CXCL10 and CXCL9
Source: PLoS Pathog. 2013 Sep 19;9(9):e1003638. doi: 10.1371/journal.ppat.1003638 (PMC3777872; doi:10.1371/journal.ppat.1003638)
Supplement: Table S2 — Expression levels of PRC2 subunits and H3K27 demethylases in wt and EBNA3A negative LCLs according to gene expression profiling data described previously. (DOCX) [file ppat.1003638.s010.docx]

**Table S2. Expression levels of PRC2 subunits and H3K27 demethylases in wt and EBNA3A negative LCLs according to gene expression profiling data described previously [1].**

| **function** | **Gene Symbol**  **(Entrez**  **Gene ID)** | **Probeset**  **(Affymetrix HG-U133A 2.0)** | **Fold-change (mean wt (n=5) / mean EBNA3A negative (n=9) LCLs)** | **Adjusted *p*-value (limma *t*-test)** | **Sum of present calls across 14 arrays** |
| --- | --- | --- | --- | --- | --- |
| **PRC2 subunits** | *EZH1* (2145) | 203249_at | 1.05 | 0.815 | 14 |
|  |  | 211310_at | - | - | 0 |
|  |  | 32259_at | 1.04 | 0.827 | 14 |
|  | *EZH2* (2146) | 203358_s_at | 0.89 | 0.689 | 14 |
|  |  | 215006_at | 0.80 | 0.193 | 14 |
|  | *EED* (8726) | 209572_s_at | 1.56 | 0.001 | 14 |
|  |  | 210656_at | 1.01 | 0.992 | 10 |
|  | *SUZ12* (23512) | 213971_s_at | 1.14 | 0.403 | 14 |
|  |  | 212287_at | 0.94 | 0.652 | 14 |
|  | *RBBP4* (5928) | 217015_at | 0.96 | 0.661 | 2 |
|  |  | 210371_s_at | 0.83 | 0.629 | 14 |
|  |  | 217301_x_at | 0.72 | 0.274 | 14 |
|  | *RBBP7* (5931) | 201092_at | 0.99 | 0.945 | 14 |
| **H3K27 demethylases** | *UTX* (7403) | 203991_s_at | 1.20 | 0.408 | 14 |
|  |  | 203992_s_at | 1.12 | 0.662 | 14 |
|  |  | 203990_s_at | 1.01 | 0.941 | 14 |
|  | *JMJD3* (23135) | 41386_i_at | 1.02 | 0.848 | 14 |
|  |  | 213146_at | 1.00 | 0.974 | 13 |
|  |  | 41387_r_at | 0.94 | 0.658 | 14 |

**1. Hertle ML, Popp C, Petermann S, Maier S, Kremmer E, et al. (2009) Differential gene expression patterns of EBV infected EBNA-3A positive and negative human B lymphocytes. PLoS Pathog 5: e1000506.**
