# Supplementary material for: Inactivation of Intergenic Enhancers by EBNA3A Initiates and Maintains Polycomb Signatures across a Chromatin Domain Encoding CXCL10 and CXCL9
Source: PLoS Pathog. 2013 Sep 19;9(9):e1003638. doi: 10.1371/journal.ppat.1003638 (PMC3777872; doi:10.1371/journal.ppat.1003638)
Supplement: Table S3 — Primers used for quantification of transcripts by qPCR. (DOCX) [file ppat.1003638.s011.docx]

**Table S3. Primers used for quantification of transcripts by qPCR.**

| **Gene** | **Primer sequence (5´-3´)** | **Annealing temp. [°C]** |
| --- | --- | --- |
| *CXCL9*-fw | GCATCATCTTGCTGGTTCTG | 60 |
| *CXCL9*-rev | TTTGGCTGACCTGTTTCTCC |  |
| *CXCL10*-fw | TGACTCTAAGTGGCATTCAAGG | 60 |
| *CXCL10*-rev | CCTTTCCTTGCTAACTGCTTTC |  |
| *CXCL11*-fw | GCCTTGGCTGTGATATTGTG | 60 |
| *CXCL11*-rev | CGATTTGGGATTTAGGCATC |  |
| *ART3*-fw | TTTCCAGGTGAAGGCTGAAG | 60 |
| *ART3*-rev | TATGCCATCAGGGCTATTCC |  |
| 18S rRNA-fw | CGGCTACCACATCCAAGGAA | 60 |
| 18S rRNA-rev | GCTGGAATTACCGCGGCT |  |
| *CDH1*-fw | GGATGTGCTGGATGTGAATG | 63 |
| *CDH1*-rev | TTAGGGCTGTGTACGTGCTG |  |
| *GIMAP4*-fw | CGGCAGTATGAGCTTCAACC | 63 |
| *GIMAP4*-rev | TGCTGCGTTTCTCACACTTC |  |
| *ADAMDEC1*-fw | AAGTGTCCCTCTGGCAGTTG | 63 |
| *ADAMDEC1*-rev | ACACGTTAGGGCTTCACAGC |  |
