# Supplementary material for: Inactivation of Intergenic Enhancers by EBNA3A Initiates and Maintains Polycomb Signatures across a Chromatin Domain Encoding CXCL10 and CXCL9
Source: PLoS Pathog. 2013 Sep 19;9(9):e1003638. doi: 10.1371/journal.ppat.1003638 (PMC3777872; doi:10.1371/journal.ppat.1003638)
Supplement: Table S4 — Antibodies used for chromatin immunoprecipitation. (DOCX) [file ppat.1003638.s012.docx]

**Table S4. Antibodies used for chromatin immunoprecipitation.**

| **Specificity, source** | **supplier** |
| --- | --- |
| α-Pol II (N-terminal), rabbit polyclonal | N-20, sc-899 X, Santa Cruz Biotechnology |
| α-H3 total, rabbit polyclonal | ab1791, abcam |
| α-H3ac, rabbit polyclonal | 06-599, Millipore |
| α-H3K4me1, rabbit polyclonal | ab8895, abcam |
| α-H3K4me3, rabbit polyclonal | 17-614, Millipore |
| α-H3K27me3, rabbit polyclonal | 17-622, Millipore |
| α-H3K27ac, rabbit polyclonal | ab4729, abcam |
| α-SUZ12, rabbit polyclonal | ab12073, abcam |
| Rabbit IgG control | Millipore |
| α-EZH2, mouse IgG2a | 17-662, Millipore |
| Mouse IgG control | Millipore |
| α-HA, rat IgG1 | High Affinity 3F10, Roche Applied Science |
| Isotype control for α-HA: α-Dog CD3 12, rat IgG1 | F1C3, E. Kremmer |
| α-EBNA2, rat IgG2a | Mixture of 1E6 and R3, E. Kremmer |
| Isotype control for α-EBNA2: α-GST, rat IgG2a | 6C9, E. Kremmer |
