# Supplementary material for: Inactivation of Intergenic Enhancers by EBNA3A Initiates and Maintains Polycomb Signatures across a Chromatin Domain Encoding CXCL10 and CXCL9
Source: PLoS Pathog. 2013 Sep 19;9(9):e1003638. doi: 10.1371/journal.ppat.1003638 (PMC3777872; doi:10.1371/journal.ppat.1003638)
Supplement: Table S5 — Primers used for qPCR quantification of DNA recovered in ChIP experiments. (DOCX) [file ppat.1003638.s013.docx]

**Table S5. Primers used for qPCR quantification of DNA recovered in ChIP experiments.**

| **Designation within manuscript** | **Position** | **Primer sequence 5´-3´** | **Annealing temp. [°C]** |
| --- | --- | --- | --- |
| A | *SDAD1* +400 | CCCATTTCTCTCTCCACTTCC | 63 |
|  |  | ACATGCCTTAGGGTTCGTTC |  |
| B | *SDAD1* TSS | CTCGTGTTTCCGGGTATGAC | 63 |
|  |  | TGAGGCTTCCGTAGCATAGC |  |
| C | Between *SDAD1* and *CXCL9* (3kb downstream of *CXCL9*) | TGTGGTAGGGTAGGAGGGAAG | 63 |
|  |  | AATCCGGTGGCTAGAGTCTG |  |
| D | *CXCL9* +500 | GCCATACATTGTGTAGCAGTC | 60 |
|  |  | GGATCCAACCAATAATCAGAG |  |
| E | *CXCL9* TSS | TGCACTCCAATCAGAACCAG | 60 |
|  |  | CCAATACAGGAGTGACTTGGAAC |  |
| F | *CXCL9* -1000 | CGGTGTGATACCACCTTACAC | 63 |
|  |  | GTTCCCTGATCACCAAGTCC |  |
| G | *CXCL9* -1700 | TTTCTCCCTGAGAGATGTAGGTG | 63 |
|  |  | AAGAAAGGAGAAGCCTGGAAG |  |
| H | *CXCL10* +1900 | TTCCCTCACCTTTCCCATC | 63 |
|  |  | GGCAGTGGAAGTCCATGAAG |  |
| I | *CXCL10* +400 | AAGGGAAGGAGGACAGAAGAG | 63 |
|  |  | CCTTAAGCACAGTTCATGTGG |  |
| J | *CXCL10* TSS | TCCCTCCCTAATTCTGATTGG | 63 |
|  |  | AGCAGAGGGAAATTCCGTAAC |  |
| K | *CXCL10* -800 | GGCACGCATAGAGACAGACC | 63 |
|  |  | ACAGTGTCTTGGAGCTGAACC |  |
| L | *CXCL10* -1600 | GAATTTGCTAGAGAGCTGAATCC | 63 |
|  |  | TGGGTTCTGTCAGTCTCTACCTC |  |
| M | *CXCL10* -2500 | AGTGATCACGTCACTCTTCTGC | 63 |
|  |  | CCAGTGTATGGGCCACTGAC |  |
| N | *CXCL11* +400 | CATGTCCACCATTTCTGTGC | 63 |
|  |  | TGTGTGCTACAGTTGTTCAAGG |  |
| O | *CXCL11* TSS | TGAGTCATGCACCTTTCCTG | 63 |
|  |  | AAGAAGGCTGGTTACCATCTG |  |
| P | *ART3* +400 | AGGGAAGACTTCTCCCTTCC | 60 |
|  |  | TACAACTATTGGCCGAATGC |  |
| Q | within *ART3* | GGCTGGGAGTACCCATGAC | 63 |
|  |  | AGGCTTTCTTCTTGGCTTGC |  |
| R | within *ART3* | AACAGCTCCAGGTCCAGTTC | 63 |
|  |  | ATCCCAAACTGTGGAAGCAG |  |
| S | *NUP54* +700 | CAGATGGAAGAAGCATGACG | 63 |
|  |  | CTGCCCATTGACTCCCTTAG |  |
| T | *NUP54* TSS | GAAACCGCCAGTCAGATCAC | 63 |
|  |  | CACAACCACCCACCTCCTAC |  |
| ctrl^ac^ | *GAPDH* TSS | TACTAGCGGTTTTACGGGCG | 63 |
|  |  | TCGAACAGGAGGAGCAGAGAGCGA |  |
| ctrl^si^ | Centromere chromosome 1 | AAGGTCAATGGCAGAAAAGGA | 63 |
|  |  | CAACGAAGGCCACAAGATGTC |  |
| R1 | Enhancer region R1 | CAGGGACGGTAAGAGCCTTC | 63 |
|  |  | AAATTCAAACAGGCCTGGAG |  |
| R2 | Enhancer region R2 | TTAGCAAGGGTGGACGGTAG | 63 |
|  |  | TCACAAGGCACTTCATCGTC |  |
| R3 | Enhancer region R3 | GTGTTTGCTCAAGGCCCTAC | 63 |
|  |  | TGCTTGCAGGGAAGGATATAAG |  |
| *CDH1* | *CDH1* TSS | GTGAACCCTCAGCCAATCAG | 63 |
|  |  | TCACAGGTGCTTTGCAGTTC |  |
| *GIMAP4* | *GIMAP4* TSS | TTGGACAGCACAGAACAACC | 60 |
|  |  | AGAGGAAGTGAGGGGAGGAG |  |
| *ADAMDEC1* | *ADAMDEC1* TSS | CCCCAATCTCACACGAAAAG | 63 |
|  |  | AAGTTGTGGTCTCCCCAGTG |  |
