# Supplementary material for: Inactivation of Intergenic Enhancers by EBNA3A Initiates and Maintains Polycomb Signatures across a Chromatin Domain Encoding CXCL10 and CXCL9
Source: PLoS Pathog. 2013 Sep 19;9(9):e1003638. doi: 10.1371/journal.ppat.1003638 (PMC3777872; doi:10.1371/journal.ppat.1003638)
Supplement: Text S1 — Bioinformatics analysis and ENCODE data sets. (DOCX) [file ppat.1003638.s014.docx]

**Text S1. Bioinformatics analysis and ENCODE data sets.**

EBNA3A repressed genes were filtered from previous gene expression analysis [[1](#_ENREF_1)] by applying different thresholds for fold-change (fc), present call (pc) and *p*-value criteria (*p*). Enrichment of H3K27me3 was analyzed for a set of 125 EBNA3A repressed genes (fc≥2.0, pc≥4, *p*≤0.05), which are recorded as NM genes by NCBI RefSeq. Genomic sections from position -500 bp relative to the TSS till the end of the respective genes were screened for H3K27me3 positive segments as defined by the Scripture algorithm [[2](#_ENREF_2)]. The H3K27me3 ChIP-seq data used for this analysis were generated in wt LCLs (GM12878) or in the EBV negative cell lines H1-hESC, HMEC, HSMM, HUVEC, K562, NHEK and NHLF [[3](#_ENREF_3)] and accessed via the UCSC genome browser [[4](#_ENREF_4)] (broadpeak files, GRCh37/hg19, <http://genome.ucsc.edu/>). Odds ratios were calculated with respect to the H3K27me3 status of the total number of genes analyzed on Affymetrix HG-U133A 2.0 arrays and tested for statistical significance using Fisher’s exact test (one-sided).

To analyze the genomic neighborhood of EBNA3A repressed genes, genomic locations for all RefSeq transcripts represented on the Affymetrix arrays were downloaded from the UCSC genome browser. Then, they were scanned chromosome by chromosome in a strand unspecific manner and consecutive transcripts fulfilling certain thresholds were counted. Overlapping transcripts were treated as one and all of them had to fulfill the given thresholds in order to be counted. Statistical significance was tested under the following Null hypothesis: Given k transcripts fulfilling the given thresholds and n transcripts in total, the probability of observing i consecutive transcripts is *p* = ${\frac{k}{n}}^{i}$. Thus, under the Null hypothesis, the number of clusters follows a binomial distribution with parameters *p* and n. Significance was also checked using a randomization experiment, where the k transcripts fulfilling the thresholds were reassigned to random transcripts and the average number of clusters and the standard deviation was determined. For the stringent thresholds from above (fc≥2.0, pc≥4, *p*≤0.05), a cluster of two consecutive transcripts occurred eleven times as compared to less than one on average for the randomization experiment (*p*=1.68e-8). For a more comprehensive analysis of the genomic neighborhoods, thresholds for filtering potential EBNA3A repressed targets were lowered. The analysis of the genomic positioning of 194 genes repressed by EBNA3A at least 1.5-fold (fc≥1.5, pc≥4, *p*≤0.05) showed that 36 of them were located in clusters of 2 co-regulated genes (*p*=2e-9, binomial test). Further lowering of thresholds (fc≥2.0, pc≥3, *p*≤1) resulted in a group of 220 EBNA3A repressed genes with 47 of them forming 17 (26), 3 (5), and 1 clusters of 2, 3 or 4 co-regulated genes, respectively (see Figure 1D).

ENCODE data for GM12878 cells displayed in Figure 2A, 3A and 7 were accessed via the UCSC genome browser on genome assembly NCBI36/hg18 (H3K27me3, H3K4me1, H3K27ac, Pol II, DNase FD and chromatin state segmentation) or GRCh37/hg19 (p300). H3K27me3, H3K4me1 and H3K27ac ChIP-seq data were retrieved from the Broad Histone track that was generated in the Bradley E. Bernstein lab [[5-7](#_ENREF_5)]. Chromatin state segmentation data [[3](#_ENREF_3),[8](#_ENREF_8)] were produced in [Manolis Kellis's Computational Biology group](http://compbio.mit.edu/#_blank) using ChIP-seq data from the Broad Histone track. DNase-seq data were generated by the Duke/UNC/UT-Austin/EBI ENCODE group. ChIP-seq data for Pol II and p300 (SC-584) were retrieved from the ENCODE TFBS track generated by the labs of [Michael Snyder](http://snyderlab.stanford.edu/), [Mark Gerstein](http://bioinfo.mbb.yale.edu/), [Sherman Weissman](http://info.med.yale.edu/bcmm/SMW/SMWhome2.html), [Peggy Farnham](http://www.genomecenter.ucdavis.edu/farnham/) and [Kevin Struhl](http://struhl.med.harvard.edu/). Genomic regions were screened for CBF1 consensus binding sites (GTGG/AGAA) [[9](#_ENREF_9)] using UCSC short match track.

**References**

**1. Hertle ML, Popp C, Petermann S, Maier S, Kremmer E, et al. (2009) Differential gene expression patterns of EBV infected EBNA-3A positive and negative human B lymphocytes. PLoS Pathog 5: e1000506.**

**2. Guttman M, Garber M, Levin JZ, Donaghey J, Robinson J, et al. (2010) Ab initio reconstruction of cell type-specific transcriptomes in mouse reveals the conserved multi-exonic structure of lincRNAs. Nat Biotechnol 28: 503-510.**

**3. Ernst J, Kheradpour P, Mikkelsen TS, Shoresh N, Ward LD, et al. (2011) Mapping and analysis of chromatin state dynamics in nine human cell types. Nature 473: 43-49.**

**4. Rosenbloom KR, Sloan CA, Malladi VS, Dreszer TR, Learned K, et al. (2013) ENCODE data in the UCSC Genome Browser: year 5 update. Nucleic Acids Res 41: D56-63.**

**5. Bernstein BE, Kamal M, Lindblad-Toh K, Bekiranov S, Bailey DK, et al. (2005) Genomic maps and comparative analysis of histone modifications in human and mouse. Cell 120: 169-181.**

**6. Bernstein BE, Mikkelsen TS, Xie X, Kamal M, Huebert DJ, et al. (2006) A bivalent chromatin structure marks key developmental genes in embryonic stem cells. Cell 125: 315-326.**

**7. Mikkelsen TS, Ku M, Jaffe DB, Issac B, Lieberman E, et al. (2007) Genome-wide maps of chromatin state in pluripotent and lineage-committed cells. Nature 448: 553-560.**

**8. Ernst J, Kellis M (2010) Discovery and characterization of chromatin states for systematic annotation of the human genome. Nat Biotechnol 28: 817-825.**

**9. Kovall RA, Hendrickson WA (2004) Crystal structure of the nuclear effector of Notch signaling, CSL, bound to DNA. Embo J 23: 3441-3451.**
