# Supplementary material for: Inactivation of Intergenic Enhancers by EBNA3A Initiates and Maintains Polycomb Signatures across a Chromatin Domain Encoding CXCL10 and CXCL9
Source: PLoS Pathog. 2013 Sep 19;9(9):e1003638. doi: 10.1371/journal.ppat.1003638 (PMC3777872; doi:10.1371/journal.ppat.1003638)
Supplement: Text S2 — Establishment of EBNA3A conditional B cell lines. (DOCX) [file ppat.1003638.s015.docx]

**Text S2. Establishment of EBNA3A conditional B cell lines.**

EBV positive and negative B cell lines conditional for EBNA3A expression were established by transfection with pRTS-1 derivatives [[1](#_ENREF_1)], which carried a puromycin resistance gene for selection of stable cell lines. 1 x 10^7^ cells were transfected with 15 µg of the respective pRTS-1 derivative by electroporation using a Gene Pulser II (Bio-Rad). Cells were allowed to recover for 24 h, platted into 48-well plates and stably selected with increasing amounts of puromycin (final concentration 1 µg/ml). Simultaneous expression of EBNA3A and NGFR was induced via treatment with 100 ng/ml Dox (Sigma-Aldrich) if not indicated otherwise. NGFR expression was quantified by flow cytometry while expression of EBNA3A was analyzed on transcript and protein level by qPCR and western blotting, respectively. Since the inducibility of pRTS-1 transfected cell lines can decrease over time [[1](#_ENREF_1)], full responsiveness to Dox was ensured for all cell lines in all experiments by flow cytometric analysis of NGFR expression. Cell lines with reduced responsiveness to Dox were positively selected for NGFR expression 14 h post Dox treatment by magnetic cell sorting using mouse α-human NGFR antibody (HB8737, ATCC) and α-mouse IgG MicroBeads (Miltenyi Biotech) according to the manufacturer´s protocol. NGFR positive cells were subsequently re-seeded in Dox containing medium until cells were further processed at the indicated points in time. The purity of the sorted NGFR positive cells was controlled by flow cytometry and always exceeded 98% of the whole cell population.

**References**

**1. Bornkamm GW, Berens C, Kuklik-Roos C, Bechet JM, Laux G, et al. (2005) Stringent doxycycline-dependent control of gene activities using an episomal one-vector system. Nucleic Acids Res 33: e137.**
