# Supplementary material for: Inactivation of Intergenic Enhancers by EBNA3A Initiates and Maintains Polycomb Signatures across a Chromatin Domain Encoding CXCL10 and CXCL9
Source: PLoS Pathog. 2013 Sep 19;9(9):e1003638. doi: 10.1371/journal.ppat.1003638 (PMC3777872; doi:10.1371/journal.ppat.1003638)
Supplement: Text S3 — Chromatin immunoprecipitation and analysis of recovered DNA. (DOCX) [file ppat.1003638.s016.docx]

**Text S3. Chromatin immunoprecipitation and analysis of recovered DNA.**

ChIP analysis was essentially performed as described [[1](#_ENREF_1)] with minor modifications. Briefly, 4x10^7^ cells (for ChIP of histones and Pol II) or 1x10^8^ cells (for ChIP of HA-EBNA3A, EBNA2, SUZ12 and EZH2) were washed twice in ice cold PBS, resuspended in 20 ml RPMI 1640 and cross-linked with formaldehyde (1% final) for 7 min at RT. The reaction was stopped by addition of glycine to a final concentration of 125 mM and gentle shaking for 5 min at RT. Cells were pelleted and washed twice in ice cold PBS. Nuclei were isolated by washing the cells 3 x with 10 ml of ice cold lysis buffer (10 mM Tris-HCl, pH 7.5, 10 mM NaCl, 3 mM MgCl_2_, 0.5% NP-40, supplemented with 1x proteinase inhibitor cocktail (PIC, Roche)) and subsequent centrifugation (300g for 10 min at 4°C). Nuclei were resuspended in 2 ml MNase digestion buffer (10 mM Tris-HCl, pH 7.5, 10 mM NaCl, 3 mM MgCl_2_, 1mM CaCl_2_, 4% NP-40, 1x PIC) and incubated with 10 or 25 U MNase S7 (Roche) at 37°C for 10 min. The reaction was stopped by addition of EGTA pH 8.0 to a final concentration of 3 mM. After addition of SDS (1% final) and NaCl (200 mM final) chromatin was sheared to an average size of 500 bp by three rounds of sonication for 6 min (5 sec pulse, 25 sec pause) at 35% amplitude using a Branson Sonifier W-250 D. Sheared chromatin was microfuged at maximum speed for 15 min at 4°C and supernatants were stored at -80°C or directly used for IP. For preparation of input DNA 12 µl aliquots (1/10^th^ of the amount used per IP) were saved at -80°C. For IPs 120 µl chromatin (~2x10^6^ or ~5x10^6^ cells of starting amount) were adjusted to 1.2 ml with IP dilution buffer (20 mM Tri-HCl, pH 8.0, 2 mM EDTA, pH 8.0, 1% Triton X-100, 150 mM NaCl, 1 x PIC) and incubated with 5 µg of antibody or 100 µl of hybridoma supernatant on a rotating platform at 4°C overnight. Antibodies used for ChIP are listed in supplementary Table S4. Protein A or G sepharose (GE Healthcare) was equilibrated with IP dilution buffer, added to the lysate and incubated at 4°C for 4 h with constant rotation. Beads were extensively washed with 2 x wash buffer I (20 mM Tris-HCl, pH 8.0, 2 mM EDTA, pH 8.0, 1% Triton X-100, 150 mM NaCl, 0.1% SDS, 1 x PIC), 1 x wash buffer II (20 mM Tris-HCl, pH 8.0, 2 mM EDTA, pH 8.0, 1% Triton X-100, 500 mM NaCl, 0.1% SDS, 1 x PIC), 1x wash buffer III (10 mM Tris-HCl, pH 8.0, 2 mM EDTA, pH 8.0, 250 mM LiCl, 1% NP-40, 1% sodium deoxycholate, 1 x PIC) for 5 min under rotation and washed twice with TE (10 mM Tris-HCl, pH 8.0, 1 mM EDTA, pH 8.0, 1 x PIC). Protein-DNA complexes were eluted with 2 x 150 µl elution buffer (25 mM Tris-HCl, pH 7.5, 10 mM EDTA, pH 8.0, 0.5% SDS) at 65°C for 15 min. Input samples were adjusted to 300 µl with elution buffer. Eluates and input samples were incubated with Proteinase K (1.5 µg/µl final, Roche) for 1 h at 42°C. Cross-linking was reversed by incubation at 65°C overnight. DNA was recovered by phenol/chloroform extraction and ethanol precipitation in the presence of glycogen or by using a QIAquick PCR purification kit (Qiagen).

The DNA amount in input samples and after IP with specific antibody or an unspecific isotype-matched IgG control was quantified by qPCR using primers listed in supplementary Table S5. To account for differences in amplification efficiencies a standard curve was generated for each primer pair using serial dilutions of sheared DNA (input) as template. DNA quantities detected in input samples were adjusted to the amount of chromatin used per IP by multiplication with 10. Values obtained from IP samples with unspecific IgG control were subtracted from the DNA amounts recovered by IP with specific antibody. The percent of input was calculated as (DNA from specific IP corrected for IgG control background/ DNA input) x 100.

**References**

**1. Ciccone DN, Morshead KB, Oettinger MA (2004) Chromatin immunoprecipitation in the analysis of large chromatin domains across murine antigen receptor loci. Methods Enzymol 376: 334-348.**
